# Exposure to 1-Butanol Exemplifies the Response of the Thermoacidophilic Archaeon Sulfolobus acidocaldarius to Solvent Stress

**DOI:** 10.1128/AEM.02988-20

**Published:** 2021-05-11

**Authors:** Jens C. Benninghoff, Laura Kuschmierz, Xiaoxiao Zhou, Andreas Albersmeier, Trong Khoa Pham, Tobias Busche, Phillip C. Wright, Jörn Kalinowski, Kira S. Makarova, Christopher Bräsen, Hans-Curt Flemming, Jost Wingender, Bettina Siebers

**Affiliations:** a Molecular Enzyme Technology and Biochemistry (MEB), Environmental Microbiology and Biotechnology (EMB), Centre for Water and Environmental Research (CWE), University of Duisburg-Essen, Essen, Germany; b Microbial Genomic and Biotechnology, Center for Biotechnology (CeBiTec), Bielefeld University, Bielefeld, Germany; c Department of Chemical and Biological Engineering, the University of Sheffield, Sheffield, United Kingdom; d National Center for Biotechnology Information, National Library of Medicine, National Institutes of Health, Bethesda, Maryland, USA; e Aquatic Microbiology, Environmental Microbiology and Biotechnology (EMB), Centre for Water and Environmental Research (CWE), University of Duisburg-Essen, Essen, Germany; f Singapore Center for Environmental Life Science Engineering (SCELSE), Nanyang Technological University, Singapore, Republic of Singapore; g Water Academy, Friedrichshafen, Germany; Kyoto University

**Keywords:** *Archaea*, extremophiles, *Sulfolobus acidocaldarius*, stress response, organic solvent, 1-butanol, biofilm, extracellular polymeric substances

## Abstract

Sulfolobus acidocaldarius is a thermoacidophilic crenarchaeon with optimal growth at 80°C and pH 2 to 3. Due to its unique physiological properties, allowing life at environmental extremes, and the recent availability of genetic tools, this extremophile has received increasing interest for biotechnological applications. In order to elucidate the potential of tolerating process-related stress conditions, we investigated the response of S. acidocaldarius toward the industrially relevant organic solvent 1-butanol. In response to butanol exposure, biofilm formation of S. acidocaldarius was enhanced and occurred at up to 1.5% (vol/vol) 1-butanol, while planktonic growth was observed at up to 1% (vol/vol) 1-butanol. Confocal laser-scanning microscopy revealed that biofilm architecture changed with the formation of denser and higher tower-like structures. Concomitantly, changes in the extracellular polymeric substances with enhanced carbohydrate and protein content were determined in 1-butanol-exposed biofilms. Using scanning electron microscopy, three different cell morphotypes were observed in response to 1-butanol. Transcriptome and proteome analyses were performed comparing the response of planktonic and biofilm cells in the absence and presence of 1-butanol. In response to 1% (vol/vol) 1-butanol, transcript levels of genes encoding motility and cell envelope structures, as well as membrane proteins, were reduced. Cell division and/or vesicle formation were upregulated. Furthermore, changes in immune and defense systems, as well as metabolism and general stress responses, were observed. Our findings show that the extreme lifestyle of S. acidocaldarius coincided with a high tolerance to organic solvents. This study provides what may be the first insights into biofilm formation and membrane/cell stress caused by organic solvents in S. acidocaldarius.

**IMPORTANCE** *Archaea* are unique in terms of metabolic and cellular processes, as well as the adaptation to extreme environments. In the past few years, the development of genetic systems and biochemical, genetic, and polyomics studies has provided deep insights into the physiology of some archaeal model organisms. In this study, we used S. acidocaldarius, which is adapted to the two extremes of low pH and high temperature, to study its tolerance and robustness as well as its global cellular response toward organic solvents, as exemplified by 1-butanol. We were able to identify biofilm formation as a primary cellular response to 1-butanol. Furthermore, the triggered cell/membrane stress led to significant changes in culture heterogeneity accompanied by changes in central cellular processes, such as cell division and cellular defense systems, thus suggesting a global response for the protection at the population level.

## Abstract (precis)

*Archaea* are unique in terms of metabolic and cellular processes, as well as the adaptation to extreme environments. In the past few years, the development of genetic systems and biochemical, genetic, and polyomics studies has provided deep insights into the physiology of some archaeal model organisms.

## INTRODUCTION

*Archaea* are widely distributed in natural environments (1). Most cultivated *Archaea* are extremophiles that thrive at environmental extremes, such as high temperatures, pH values, high salt concentrations, or combinations thereof (2). In particular, thermophiles and hyperthermophiles, with growth optima above 60°C and 80°C, respectively, are of interest for biotechnological applications in high-temperature industrial processes (3, 4). They are able to produce enzymes (extremozymes/thermozymes) that are functional under extreme conditions because of enhanced enzyme rigidity and stability, and they have been shown to be active in organic solvents and ionic liquids (5). In addition, *Archaea* possess a unique membrane lipid composition. In contrast to *Bacteria* and *Eukarya*, they use isoprenoid hydrocarbon side chains linked to *sn*-glycerol-1-phosphate via ether linkage, forming monopolar diether lipids (archaeol) or membrane-spanning bipolar tetraether lipids (caldarchaeol) (6). These archaeal membranes are more stable against stressors (7).

One promising platform organism for biotechnology is the thermoacidophilic crenarchaeon Sulfolobus acidocaldarius (3, 4, 8, 9). S. acidocaldarius is an obligately aerobic organism growing optimally under the two extreme conditions of low pH values (2.0 to 3.5) and high temperatures (75°C to 80°C). The species is genetically tractable (10), enabling metabolic engineering for potential applications in industrial processes (4). S. acidocaldarius is able to form biofilms (11, 12), defined as microbial aggregates embedded in a matrix of extracellular polymeric substances (EPS) on surfaces and other interfaces (13). Proteins, carbohydrates, and DNA have been identified as constituents of the EPS matrix of S. acidocaldarius (14). The biofilm mode of life is dominant among prokaryotic microorganisms (15) and offers advantages for survival compared to the planktonic lifestyle, for example, an enhanced tolerance against adverse environmental conditions (13) that may be encountered in biotechnological processes due to toxic reactants or products.

1-Butanol is a key commodity widely used as a solvent or chemical feedstock. So far, 1-butanol is mainly produced chemically by the Oxo process (16). Human dependence on petroleum-derived fuels, the corresponding depletion of fossil resources, and emission of greenhouse gases, particularly CO_2_, promoted the search for more environmentally friendly alternatives. In this context, biobutanol represents a promising alternative as a fuel additive and biofuel for direct replacement of gasoline (17, 18). Production of biobutanol from renewable resources is predominantly accomplished by *Clostridium* strains via acetone butanol ethanol (ABE) fermentation (16). However, while ABE fermentation provided approximately 66% of the world’s supply of 1-butanol until the 1950s, bio-based butanol production was outcompeted by petroleum-based processes after this period (16).

A problem in the production of biobutanol is its toxicity toward microbial cells. For a vast majority of microorganisms, a growth limit at 1% to 2% (vol/vol) 1-butanol in nutrient medium has been observed in liquid cultures (19, 20). There is the widely accepted notion that 1-butanol toxicity results from its chaotropic effects on the cytoplasmic cell membrane, leading to the disruption of nutrient and ion transport and the loss of the membrane potential (21, 22). Bacteria and eukaryotic microorganisms are able to adapt to the presence of aliphatic, toxic alcohols, including acetone, ethanol, butanol, isobutanol, and propanol, with the development of an enhanced tolerance, allowing survival and growth at elevated concentrations of these compounds (20, 23, 24). The adaptation strategies are versatile (21, 22, 25). Microorganisms can respond to alcohol exposure by changing their membrane lipid composition to sustain membrane fluidity, called “homeoviscous adaptation” (26). This process may include a shift in the ratio of unsaturated to saturated lipids, branched and unbranched lipids, and/or a change in isomerization and cyclization state or headgroup composition (20, 22, 27). In Gram-negative bacteria, outer membrane modifications (e.g., alterations in the lipopolysaccharide content and porin expression, increased interactions with divalent metal ions for membrane stabilization, and outer membrane vesicle formation) have been reported (27). Other cellular responses described for bacteria include the upregulation of energy-dependent efflux systems to reduce the intracellular solvent concentration and the metabolic degradation of solvents (28). Organic solvents, including 1-butanol, were shown to enhance expression of heat shock proteins, including molecular chaperones that assist correct protein folding and transport, as well as the recycling of defective proteins (29). Cell aggregation and biofilm formation were shown to enhance tolerance to 1-butanol, as observed for the butanol production strain Clostridium acetobutylicum (30). Further, changes in the composition of EPS were reported for biofilms of C. acetobutylicum and Pseudomonas taiwanensis (31, 32).

Regarding organic solvent tolerance, archaeal extremophiles may offer advantages over mesophilic organisms due to their intrinsic robustness and adaptation to hostile environments (2–4, 9). This study aims to fill this gap in knowledge by investigating the natural ability of the thermoacidophilic crenarchaeon S. acidocaldarius, as an archaeal model organism, to tolerate 1-butanol and its cellular response toward this industrially relevant organic solvent.

## RESULTS

### Effect of 1-butanol on cell growth in liquid cultures.

**FIG 1:**
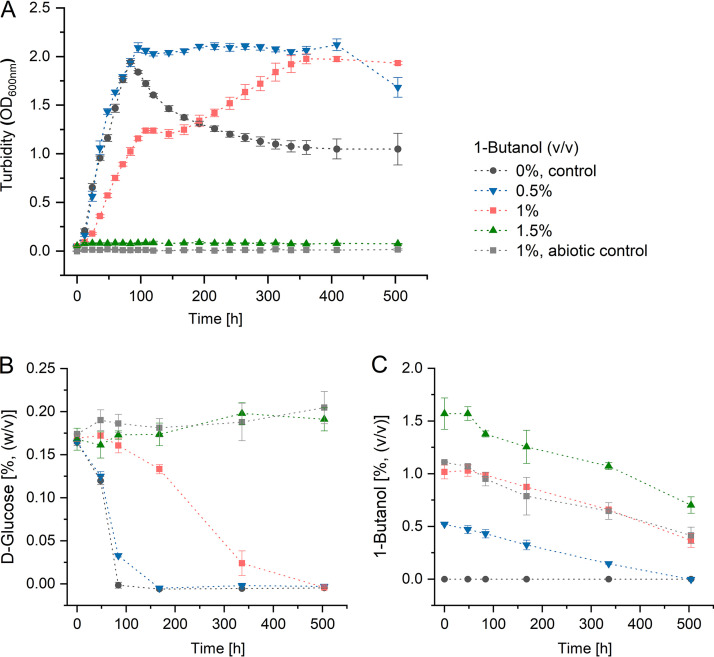
Effect of 1-butanol on cell growth of S. acidocaldarius DSM 639 in liquid cultures. S. acidocaldarius cells were grown in Brock medium supplemented with 0.1% (wt/vol) NZ-amine and 0.2% (wt/vol) d-glucose in the absence and presence of 1-butanol (0 to 1.5% [vol/vol], long-neck flasks, 76°C, pH 3.0, 180 rpm). An abiotic control medium with 1% (vol/vol) 1-butanol was used to monitor 1-butanol loss due to evaporation. (A) Growth of S. acidocaldarius determined by turbidity measurement (OD_600_). (B) d-Glucose consumption. (C) Change of 1-butanol concentration. Experiments were carried out in four biological replicates.

Initially, the effect of 1-butanol on S. acidocaldarius was investigated using liquid cultures. Growth (as measured by optical density at 600 nm [OD_600_]), d-glucose consumption, and 1-butanol concentration were determined throughout the incubation period of 3 weeks (Fig. 1). The levels of growth of S. acidocaldarius at 76°C in the absence and presence of 0.5% (vol/vol) (4.05 g/liter; 55 mM) 1-butanol were similar, reaching maximum OD_600_ values after 84 h of cultivation (Fig. 1A). On further incubation, cultures without 1-butanol showed a significant decrease of OD_600_ values, while OD_600_ values of cultures with 0.5% (vol/vol) 1-butanol remained unchanged for a prolonged time period of approximately 312 h (Fig. 1A). Cells exposed to 1% (vol/vol) (8.10 g/liter; 109 mM) 1-butanol showed biphasic growth and reached the stationary phase with a considerable delay (residual growth rate of 52%, 0 to 72 h) compared to cells grown without or with 0.5% (vol/vol) 1-butanol. Concomitantly, S. acidocaldarius showed a substantial delay in d-glucose utilization when exposed to 1% (vol/vol) 1-butanol (Fig. 1B). Neither growth nor glucose degradation was observed in planktonic S. acidocaldarius cultures supplemented with 1.5% (vol/vol) (12.15 g/liter; 164 mM) 1-butanol (Fig. 1A and B). The concentration of 1-butanol decreased at similar rates in all cultures, including a cell-free abiotic control with 1% (vol/vol) 1-butanol (Fig. 1C).

Culturability of S. acidocaldarius was examined using spot plates (Fig. S1 in the supplemental material). Samples of cell cultures were taken at different time points, diluted, and spotted on Brock-Gelrite plates, followed by incubation of the plates at 76°C for 4 days. Cells from cultures without 1-butanol, as well as with 0.5 and 1% (vol/vol) 1-butanol, were able to grow on the spot plates, while complete inhibition of culturability was observed for cells from cultures with 1.5% (vol/vol) 1-butanol (Fig. S1).

“Collars” of a slimy material developed on the inner glass surfaces of the flasks at the air-liquid interface of liquid cultures exposed to 0.5 and 1% (vol/vol) 1-butanol within 1 week of incubation and remained visible throughout the 3-week incubation period (Fig. S2). Crystal violet staining improved the visibility of the slimy material attached to the glass surface (Fig. S2C). Microscopic examination of the slime revealed high numbers of cells densely packed and embedded in the slime matrix, indicating the formation of biofilms upon exposure to subinhibitory concentrations of 1-butanol (Fig. S2B). Debris of biofilms were observed in S. acidocaldarius cultures grown without 1-butanol, while no biofilm was formed by cells exposed to 1.5% (vol/vol) 1-butanol (Fig. S2C). Inspection of culture liquid (planktonic cells) by means of phase-contrast microscopy revealed both single cells and cell aggregates in the log growth phase (OD_600_ values between 0.5 and 1.5) independent of the absence or presence of 0.5 and 1% (vol/vol) 1-butanol (Fig. S3). At 1.5% (vol/vol) 1-butanol without any growth, cell aggregates were not observed in the culture medium.

To investigate the specificity of the cell response to 1-butanol, S. acidocaldarius was grown in the presence of other short-chain alcohols, namely, ethanol, 1-propanol, and isobutanol (liquid cultures). The formation of biofilms at the solid-air-liquid interphase was observed for ethanol at 1 to 4% (vol/vol), 1-propanol at 0.5 to 2.5% (vol/vol), and isobutanol at 0.5% and 1% (vol/vol) of the corresponding alcohol added to the culture medium (Fig. S4A). Thus, the increase of biofilm amounts at the solid-air-liquid interphase seems to be a general response of S. acidocaldarius to short-chain alcohols.

### Effect of 1-butanol on biofilm formation and architecture.

**FIG 2:**
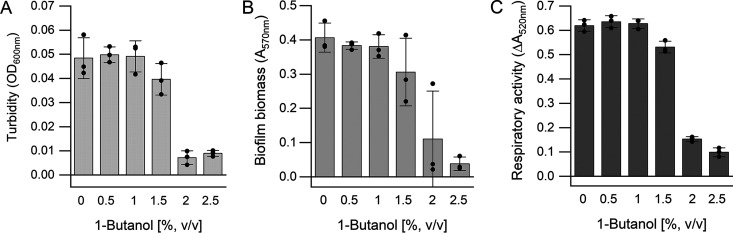
Concentration-dependent effect of 1-butanol on biofilm formation and cell viability of S. acidocaldarius. Cells were statically incubated in 96-well microtiter plates in Brock medium containing 0.1% (wt/vol) NZ-amine and 0.2% (wt/vol) d-glucose in the presence of different 1-butanol concentrations (0 to 2.5% [vol/vol]) at 76°C for 4 days. (A) OD_600_ values of biofilm cells. (B) Quantification of biofilm biomass by crystal violet staining (absorbance at 570 nm). (C) Respiratory activity of S. acidocaldarius biofilm cells determined by the resazurin assay, measuring resazurin reduction by absorbance at 520 nm. Activity is expressed as the decrease of absorbance over 3 h, ΔA_520_. Experiments were carried out in three biological replicates. Data points are indicated as closed circles in the diagram.

Biofilm formation was quantified by a commonly used microtiter plate biofilm assay. S. acidocaldarius was cultivated in 96-well polystyrene microtiter plates with different 1-butanol concentrations (0%, 0.5%, 1%, 1.5%, 2%, and 2.5% [vol/vol] 1-butanol) under static conditions at 76°C for 4 days. Growth was determined by turbidity measurements (OD_600_) (Fig. 2A), and biofilm biomasses were quantified based on crystal violet staining of surface-attached biomass (Fig. 2B). Respiratory activity of biofilms was determined using an adapted cell viability assay based on the reduction of resazurin (Fig. 2C). The turbidity values (OD_600_) of suspended biofilm cells remained nearly constant up to 1.5% (vol/vol) 1-butanol (Fig. 2A). At 2% (16.2 g/liter; 218 mM) and 2.5% (vol/vol) (20.25 g/liter; 273 mM) 1-butanol, the turbidity values of biofilm cells decreased significantly. This coincided with drops in biofilm biomass (Fig. 2B) and respiratory activity of the biofilms (Fig. 2C). However, weak biofilm formation and low respiratory activity were still observed, indicating the presence of metabolically active cells at elevated 1-butanol concentrations of up to 2.5% (vol/vol).

**FIG 3:**
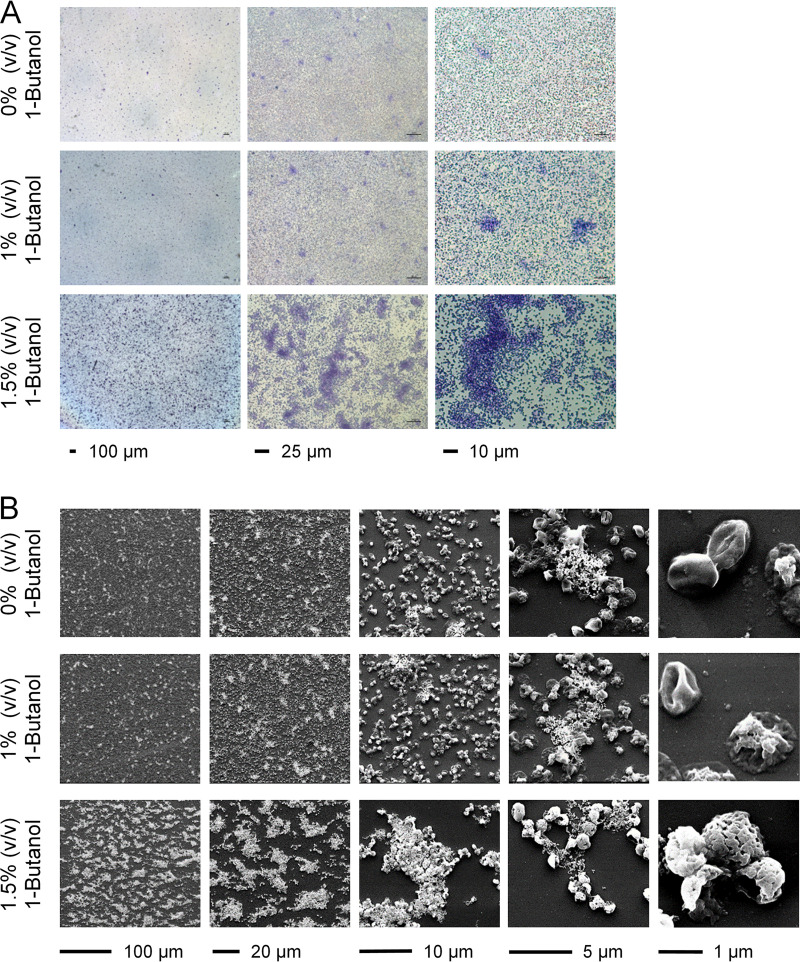
Effect of 1-butanol on S. acidocaldarius cell distribution and morphology analyzed by light microscopy (A) and scanning electron microscopy (B). S. acidocaldarius was grown on glass surfaces for 4 days at 76°C in the absence and presence of 1% and 1.5% (vol/vol) 1-butanol. (A) Attached cells stained with crystal violet and air dried for subsequent analysis by light microscopy. (B) Visualization of biofilm cell distribution and morphology by SEM.

Different microscopic techniques were used to visualize the distribution, architecture, and EPS of S. acidocaldarius biofilms directly on surfaces in the absence and presence of 1-butanol. First, we analyzed the distribution of crystal violet-stained biofilm cells on glass slides in combination with light microscopy. After growth for 4 days, a change in the pattern of cell distribution depending on the 1-butanol concentration was observed (Fig. 3A). In the absence of 1-butanol, the cells were homogeneously distributed on the glass surface predominantly as a single layer of cells, occasionally interspersed with cell aggregates (microcolonies) (Fig. 3A). Under the influence of 1% (vol/vol) 1-butanol, a higher occurrence of microcolonies was observed (Fig. 3A). Exposure to 1.5% (vol/vol) 1-butanol resulted in the formation of more-pronounced microcolonies with an irregular pattern of distribution (Fig. 3A). Similar to the results for 1-butanol, the formation of S. acidocaldarius microcolonies on glass substratum was also observed when the cells were exposed to certain solvent-specific concentrations of ethanol, 1-propanol, and isobutanol (Fig. S4B).

In accordance with light microscopy, scanning electron microscopy (SEM) images showed the formation of microcolonies of S. acidocaldarius that developed in the presence of 1% and 1.5% (vol/vol) 1-butanol (Fig. 3B). Cell aggregates were partially surrounded by extracellular material, probably comprising EPS (Fig. 3B). Thus, these images confirmed that 1-butanol promoted the formation of cell aggregates with concomitant production of extracellular components. The cell shape also changed with increasing 1-butanol concentrations. In the absence of 1-butanol and in the presence of 1% (vol/vol) 1-butanol, the cells showed two types of morphology: (i) lobe-shaped with a smooth surface structure and a size of approximately 1 μm, as previously described by Brock et al. (8), and (ii) flat cells with a more irregular structure (Fig. 3B). In the presence of 1.5% (vol/vol) 1-butanol, a third cell morphotype was observed, where the surface of S. acidocaldarius cells appeared more rounded with a perforated surface structure (Fig. 3B).

**FIG 4:**
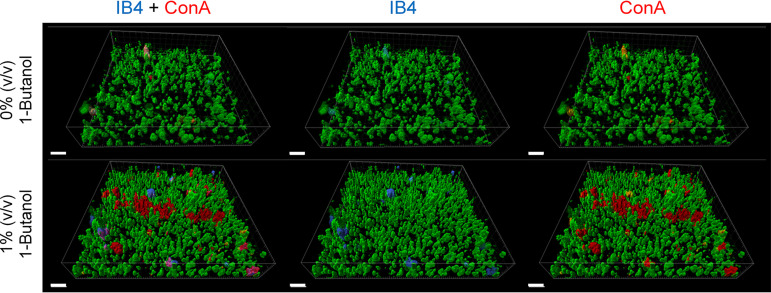
Effect of 1-butanol on biofilm architecture of S. acidocaldarius analyzed by confocal laser scanning microscopy. Submersed biofilms were grown at 76°C for 4 days under static conditions in μ-dishes (ibidi). Cells were stained with SYTO 9 (green signals), carbohydrates were visualized using the fluorescently labeled lectins GS-IB4–Alexa 568 (binding to α-d-galactosyl and *N*-acetyl-d-galactosamine residues, blue signals) and ConA-Alexa 633 (binding to α-mannopyranosyl and α-glucopyranosyl residues, red signals). Scale bars: 10 μm.

Confocal laser scanning microscopy (CLSM) was used to visualize the three-dimensional biofilm architecture and the occurrence of extracellular carbohydrates. S. acidocaldarius was incubated at 76°C for 4 days in μ-dishes (ibidi). Submersed biofilms were analyzed by SYTO 9 staining of cells and addition of fluorescently labeled lectins GS-IB4 (an isolectin from Griffonia simplicifolia) and ConA (concanavalin A) that had previously proved suitable to visualize carbohydrates as constituents of *Sulfolobus* EPS (11). CLSM revealed that S. acidocaldarius biofilms mainly consisted of tower-like structures (Fig. 4). While only a few carbohydrate-containing structures were detected in S. acidocaldarius biofilms without 1-butanol, the presence of 1% (vol/vol) 1-butanol led to the detection of more sugar residues bound by lectins GS-IB4 and ConA (Fig. 4). Thus, the amount of carbohydrate-containing structures on the surface of S. acidocaldarius biofilms increased in response to 1-butanol exposure. Overall, the signals of ConA (Fig. 4, red) were more dominant than the signals of GS-IB4 binding (Fig. 4, blue). Some carbohydrate structures seemed to be targeted by both lectins.

### Effect of 1-butanol on EPS composition.

**FIG 5:**
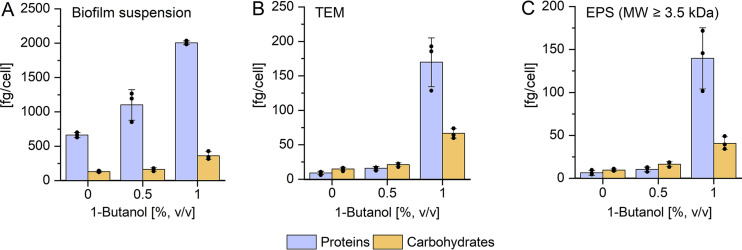
Influence of 1-butanol on extracellular polymeric substance (EPS) composition. S. acidocaldarius biofilms were incubated at 76°C for 4 days and the absolute concentration values of EPS components were normalized to the total cell counts. The amounts of proteins and carbohydrates were determined in different biofilm fractions (A to C). (A) Total biofilms suspended in phosphate buffer containing biofilm cells and extracellular compounds. (B) Total extracellular material (TEM): fractions after EPS extraction by the CER method and sterile filtration (CER-extracted EPS material, comprising high- and low-molecular-weight [MW] extracellular compounds). (C) EPS: molecules ≥3.5 kDa (EPS compounds obtained after dialysis of TEM fraction using 3.5-kDa-cutoff membranes). Experiments were carried out in three biological replicates. Data points are indicated as closed circles in the diagram.

Since microscopic analyses of S. acidocaldarius indicated the presence and enhanced formation of extracellular material due to 1-butanol exposure, EPS isolation and quantification from biofilms grown in polystyrene petri dishes at 76°C for 4 days was performed (Fig. 5, Fig. S5). The turbidity of the isolated aqueous phase (OD_600_ of planktonic cells) decreased with increasing 1-butanol concentrations (Fig. S5A). The wet weight of biofilm mass harvested from the bottom of petri dishes was the same in the absence and in the presence of 0.5 and 1% (vol/vol) 1-butanol (approximately 110 μg/cm^2^), and the biofilm cell numbers were 6.2 to 8.3 × 10^5^ cells/cm^2^ (Fig. S5). Suspensions of harvested S. acidocaldarius biofilms were used to extract EPS with a cation-exchange resin. After the extraction procedure, the supernatant was sterile filtered to obtain cell-free total extracellular material (TEM). The final EPS fraction was obtained after removal of low-molecular-weight compounds by dialysis (cutoff, 3.5 kDa) (14, 33). The amounts of carbohydrates and proteins per biofilm cell were determined in the biofilm suspensions, the TEM, and the EPS fraction (Fig. 5A to C). In biofilm suspensions, the amounts of proteins as well as carbohydrates increased with an increasing concentration of 1-butanol in the growth medium (Fig. 5A). The increase in carbohydrate and protein content in response to 1% (vol/vol) 1-butanol was even more pronounced within the TEM and EPS fractions (Fig. 5B). The carbohydrate amount per biofilm cell increased 5-fold and the amount of proteins per cell was 19-fold higher. However, 0.5% (vol/vol) 1-butanol did not cause significant increases in carbohydrates and proteins compared to the control without 1-butanol. The increases in protein and carbohydrate amounts in the total cell-free extracellular components (Fig. 5B) were due to the presence of mainly high-molecular-weight protein and carbohydrate compounds in the EPS (Fig. 5C). When exposed to 1% (vol/vol) 1-butanol, the main component of the EPS was proteins (Fig. 5C). In contrast, carbohydrates were identified as the main component of biofilm cells without and with exposure to 0.5% (vol/vol) 1-butanol (Fig. 5C).

In summary, microscopic and biochemical analyses indicated that S. acidocaldarius responded to 1-butanol exposure by a significant change in EPS composition and biofilm architecture, accompanied by an alteration in cell morphotypes. Overall, these results suggested that S. acidocaldarius might change its gene expression in response to 1-butanol concentrations above 0.5% (vol/vol). Therefore, transcriptome and initial proteome analyses were conducted to obtain insights into the cellular response toward 1-butanol.

### Genome-wide transcriptional response of S. acidocaldarius biofilms to 1-butanol exposure.

S. acidocaldarius was grown in petri dishes (static incubation) in the absence and presence of 0.5% and 1% (vol/vol) 1-butanol at 76°C for 4 days. Planktonic and biofilm cells were harvested and the transcriptional response toward 1-butanol was analyzed. Here, regulated genes with more than 4-fold changes (log_2_ fold change ≥ 2) are discussed in detail (Table 1, Table S1, Data Set S1). In response to lifestyle, i.e., biofilm (BF) versus planktonic (PL) cells, the expression of only 15 and 44 genes was significantly changed in the absence (BF0/PL0) and in the presence of 1% (vol/vol) 1-butanol (BF1/PL1), respectively, indicating that after 4 days of static growth, both biofilm and planktonic cells are quite similar with respect to their gene expression profiles (Table 1). In agreement with our previous experimental observations on growth of S. acidocaldarius with 0.5% (vol/vol) 1-butanol (Fig. 1 and 2), the transcriptional response in planktonic (PL05/PL0) and biofilm (BF05/BF0) cells toward 0.5% (vol/vol) 1-butanol was quite low (8 and 16 differentially expressed genes, respectively). Major transcriptional changes were observed in planktonic and biofilm cells in the presence of 1% (vol/vol) 1-butanol, with 122 (PL1/PL0) and 117 (BF1/BF0) differentially expressed genes, respectively (Table 1, Fig. S6). Notably, most genes were downregulated in biofilms (74 genes) and planktonic cells (89 genes). Among these differentially regulated genes, 42 genes (16 up- and 26 downregulated genes) displayed a common regulation in both biofilms and planktonic cells (Fig. S6).

**TABLE 1:** Data set correlation and number of differentially transcribed genes

Comparison		No. of regulated transcribed genes (log_2_ fold change ≥2 or ≤−2; A value ≥ 2)			
Sample type	Designator^*a*^	*R*^2^ value	Down	Up	Total
Lifestyle	BF0/PL0	0.91	13	2	15
	BF1/PL1	0.88	6	38	44
Biofilm	BF1/BF0	0.77	74	43	117
	BF05/BF0	0.91	4	12	16
	BF1/BF05	0.83	56	20	76
Planktonic	PL1/PL0	0.76	89	33	122
	PL05/PL0	0.93	3	5	8

a BF, biofilm cells; PL, planktonic cells; 0, control without 1-butanol; 05, 0.5% (vol/vol) 1-butanol; 1, 1% (vol/vol) 1-butanol.

**FIG 6:**
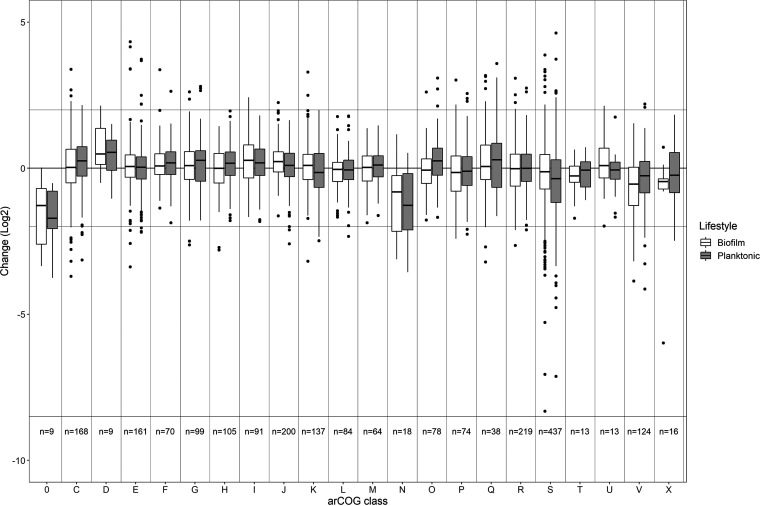
ArCOG classification of genes regulated in response to 1% (vol/vol) 1-butanol. Differential regulation (log_2_ fold change) of genes involved in the different arCOG categories in biofilm and planktonic cells of S. acidocaldarius (static cultivation in petri dishes, 76°C, 4 days) grown in the presence and absence of 1% (vol/vol) 1-butanol. ArCOG classes: 0, uncharacterized; C, energy production and conversion; D, cell cycle control, cell division, chromosome partitioning; E, amino acid transport and metabolism; F, nucleotide transport and metabolism; G, carbohydrate transport and metabolism; H, coenzyme transport and metabolism; I, lipid transport and metabolism; J, translation, ribosomal structure and biogenesis; K, transcription; L, replication, recombination and repair; M, cell wall/membrane/envelope biogenesis; N, cell motility; O, posttranslational modification, protein turnover, chaperones; P, inorganic ion transport and metabolism; Q, secondary metabolites biosynthesis, transport and catabolism; R, general function prediction only; S, function unknown; T, signal transduction mechanisms; U, intracellular trafficking, secretion, and vesicular transport; V, defense mechanisms; X, mobilome: prophages, transposons.

For most archaeal clusters of orthologous gene (arCOG) categories, no major changes in gene expression were observed for biofilm and planktonic cells in response to 1-butanol (1%) exposure (Fig. 6) (34). However, for the arCOG categories S (function unknown, *n* = 437), 0 (uncharacterized, *n* = 9), and N (cell motility, *n* = 18), a strong downregulation was observed in both planktonic and biofilm cells.

Due to the changes in cell morphology observed via SEM (Fig. 3B), we inspected the distribution of predicted transmembrane helices among these differentially regulated genes in more detail (Fig. S7), revealing that 21% and 25% of the upregulated and 42% and 52% of the downregulated genes in biofilm and planktonic cells, respectively, possess at least one transmembrane helix. For most of these predicted membrane (associated) proteins, the function is unknown. Also, the most highly downregulated genes in biofilm upon 1-butanol exposure (log_2_ fold change ≥ −4) are membrane proteins of unknown function (Table S2).

**FIG 7:**
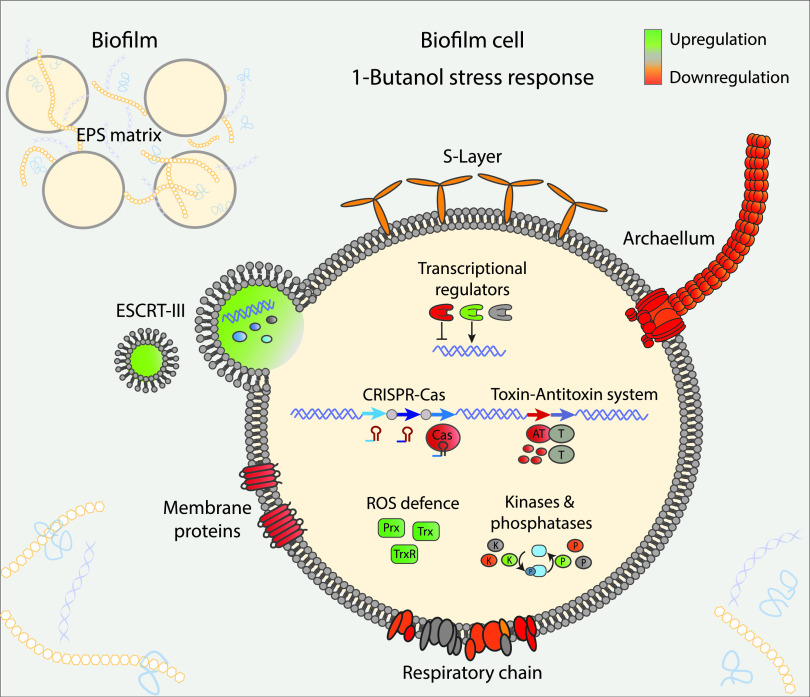
Model of 1-butanol stress response in S. acidocaldarius biofilm cells. Increased and decreased transcription of genes in cellular structures and processes are depicted by green and red color, respectively. Major EPS matrix components (polysaccharides, proteins, and eDNA) are distributed between the cells. Genes encoding membrane proteins, the archaellum for motility, the adaptive immune system (CRISPR-Cas), the dormancy- or cell death-inducing defense (toxin-antitoxin) system, and components of the respiratory chain are downregulated (red). Genes encoding proteins of the ROS defense system and the ESCRT-III system for vesicle formation and/or cytokinesis are upregulated (green). Several transcriptional regulators, as well as protein kinases (K) and protein phosphatases (P) for reversible protein phosphorylation, were differentially expressed. For detailed discussion see the text.

Based on the great overlap of the gene regulation patterns found for planktonic and biofilm cells, a detailed comparative analysis of the transcriptome data was performed, focusing on biofilm cells grown without and with 1% (vol/vol) 1-butanol. In addition to membrane proteins, genes encoding cell surface structures were strongly affected by 1-butanol exposure (Table S1). In line with the 1-butanol-enhanced biofilm formation, most genes encoding the archaellum for motility (*flaX*-*flaJ* gene cluster, *saci_1172 to saci_1178*) were significantly downregulated (Fig. 7), whereas UV-induced pili (*ups* genes) for genetic DNA exchange via conjugation and adhesive pili (*aap* genes) for cell attachment were, if at all, only slightly affected (35). *Crenarchaeota*, including *Sulfolobus* spp., rely on the endosomal sorting complexes required for transport (ESCRT III) machinery for cell division (Cdv), vesicle formation, and budding (36–39). In response to 1-butanol exposure, the gene encoding one of the three CdvB paralogs (*cdvB1*, *saci_0451*) is significantly upregulated (38, 39).

Also, for several transcriptional regulators, a differential gene expression was observed; however, only *arnR1* (PL1/PL0) was more than 2-fold downregulated (Table S1). Saci_1171 (ArnR1) is, alongside ArnR (Saci_1180), one of the positive transcriptional regulators for archaellum biosynthesis (40). For the gene encoding the archaeal biofilm regulator 1 (AbfR1; *saci_0446*), a slight upregulation (log_2_ fold change of 1.71) in response to 1-butanol was observed in biofilm cells (41, 42), whereas *saci_1223* (encoding AbfR2) was slightly downregulated (log_2_ fold change of −1.54) (43).

Notably, we observed a significant regulation of the CRISPR-Cas (CRISPR: clustered, regularly interspaced, short, palindromic repeats; Cas: CRISPR-associated) system (44, 45). In the presence of 1-butanol, a significant downregulation of the *Sulfolobus*-specific type III system genes (*saci_1893* to *saci_1899*) was observed (Table S1).

Like the CRISPR-Cas system, several genes of the toxin-antitoxin (TA) system (46) were also downregulated in S. acidocaldarius cells in response to 1-butanol exposure, with *saci_1056* (antitoxin, CopJ/RHH family) and *saci_1124* (CopG/RHH family DNA binding protein, only BF1/BF0) being the most prominent ones (Table S1). Additionally, one gene of the HEPN-MNT system (HEPN toxin and MNT [minimal nucleotide transferase] [*saci_1928*] antitoxin) showed a slight upregulation.

Concerning central metabolism, the 5-oxoprolinase, involved in the degradation of pyroglutamate, was one of the most upregulated genes in response to 1-butanol exposure (Table S1) (47). In addition, the gene cluster including *saci_2293* (2-keto-4-pentenoate hydratase/2-oxohepta-3-ene-1,7-dioic acid hydratase), *saci_2294* (aromatic ring hydroxylase), and *saci_2295* (catechol 2,3-dioxygenase or other lactoylglutathione lyase family enzyme) was also significantly upregulated in both lifestyles upon 1-butanol exposure. The three genes encode proteins involved in the catechol meta-cleavage pathway for degradation of aromatic compounds/amino acids (48).

Finally, major regulation was observed at the transcript level for the branched aerobic respiratory chain (49). Whereas the cytochrome *bc*_1_ complex (*saci_1859* to *saci_1862*) and one of the three terminal oxidases, the DoxBCE complex (*saci_0097* to *saci_0099*), were significantly downregulated in biofilm cells in response to 1-butanol, the SoxABCDL complex (*saci_2086* to *saci_2089*) was not regulated at all and the SoxEFGHIM complex (*saci_2258* to *saci_2263*) was mainly downregulated in planktonic cells upon 1-butanol exposure. Genes encoding thioredoxin (*saci_1823*) and peroxiredoxin (*saci_1125*) were slightly upregulated in biofilms exposed to 1-butanol (log_2_ fold changes of 1.4 and 1.2, respectively). Consistent with this, the gene *saci_1169*, encoding a thioredoxin reductase TrxB, catalyzing the reduction of thioredoxin, was upregulated 6.1-fold.

In addition to the transcriptional changes, the global proteome response of biofilm cells in the absence and presence of 1% (vol/vol) 1-butanol was analyzed. Planktonic cells could not be considered for further analysis since the cell mass retrieved after growth in the presence of 1% (vol/vol) 1-butanol was below the limit for analysis. The presence of 1% (vol/vol) 1-butanol resulted in the upregulation of 93 proteins (11 significant) and the downregulation of 114 proteins (23 significant) in biofilm cells (Table S3). The highest upregulation in response to 1-butanol was found for the ribosomal proteins Saci_0642 (RplA/L37e) and Saci_0583 (RpsN/S14). Consistent with the transcriptome analysis, proteins of the respiratory chain, namely, two proteins of the cytochrome bc_1_ complex (Saci_1860 and Saci_1862) and one protein subunit 1 of the terminal oxidase complex DoxBCE (Saci_0097), were significantly downregulated (Table S3). In addition, the S-layer protein SlaA was found to be downregulated in both omics analyses. However, in general, no significant overlap was observed between the transcriptome and proteome data. This discrepancy was also observed upon starvation in S. acidocaldarius (50) and it was suggested that response to stress conditions acts on multiple layers of gene expression and regulation.

## DISCUSSION

Here, we examined the effect of 1-butanol on the thermoacidophilic archaeon S. acidocaldarius to study its ability to tolerate 1-butanol as well as its response toward solvent stress.

### Butanol toxicity.

In liquid cultures, S. acidocaldarius was able to grow in the presence of 1% (vol/vol) 1-butanol without prior adaptation, while no growth was observed with 1.5% (vol/vol) 1-butanol. To date, 1-butanol tolerance has only been investigated in a few *Archaea*, including mesophiles (Natronomonas pharaonis, Halorubrum lacusprofundi, and Methanosarcina acetivorans) and hyperthermophiles (Methanocaldococcus jannaschii and Aeropyrum pernix) (20). In the presence of 0.25% (vol/vol) 1-butanol, *N. pharaonis* and *M. acetivorans* showed no growth, while *H. lacusprofundi* and *M. jannaschii* were able to grow. *A. pernix* was reported to grow in the presence of 0.5% (vol/vol) 1-butanol (20). The 1-butanol tolerance of S. acidocaldarius is in a similar range as that observed for planktonic mesophilic bacterial and yeast species commonly used as model organisms in biotechnology (1 to 2% [vol/vol]), such as Escherichia coli, Bacillus subtilis, C. acetobutylicum, and Saccharomyces cerevisiae (19, 20, 51–53).

Thus, planktonic S. acidocaldarius cells possess a 2- to 4-fold higher tolerance to 1-butanol than other archaeal organisms reported and a similar tolerance as mesophilic organisms well established in biotechnology. This is remarkable, since S. acidocaldarius was not adapted to the solvent and is challenged by both high temperature (76°C) and low pH (pH 3.0). In the future, further adaptation approaches may enhance its 1-butanol tolerance, as shown for Pseudomonas putida strains (54) and C. acetobutylicum (53, 55).

### Biofilm formation and EPS composition.

Using analytical and microscopic techniques, we demonstrated that S. acidocaldarius responded to 1-butanol exposure with enhanced biofilm formation. In the presence of 0.5% and 1% (vol/vol) 1-butanol, larger amounts of adhered cells were observed on the glass surfaces of culture flasks. In contrast, only debris of biofilm was visualized for S. acidocaldarius cultures grown without 1-butanol. Thus, the addition of 1-butanol may promote the biofilm formation or the formation of a more robust and stable biofilm that is more resistant to the shear forces in shaking cultures.

Under static cultivation conditions in microtiter plates, biofilm cells showed enhanced 1-butanol tolerance at 1.5% (vol/vol) up to 2.5% (vol/vol). However, at concentrations in the range of 1.5% to 2.5% (vol/vol) 1-butanol, biomass yield and metabolic activity of S. acidocaldarius biofilms also decreased gradually. For S. acidocaldarius, an enhanced biofilm formation was previously reported for other environmental stressors, including nonoptimal temperature (60°C, 85°C), increased pH values (pH 4 to 6), and increased pH along with higher iron concentration (pH 6.0, 0.065 g/liter iron) (11). Similarly, immobilized C. acetobutylicum biofilm cells showed increased butanol tolerance with growth at 1.5 % (vol/vol) 1-butanol and improved ABE productivity (53). While planktonic cells showed no growth at 1.5% (vol/vol) butanol, C. acetobutylicum biofilm cells showed continuous growth (53). Thus, in general, biofilm formation seems to enhance microbial tolerance to suboptimal environmental factors due to diverse protective mechanisms, commonly with a major contribution of the EPS matrix (12, 13).

In agreement with this general observation, we detected a significant increase in the amounts of EPS proteins and carbohydrates for S. acidocaldarius biofilms at 1% (vol/vol) 1-butanol. These EPS components may facilitate enhanced biofilm adhesion to the solid surface and cohesion of cells inside the biofilm. Consistently, CLSM analysis confirmed an increased concentration and changed composition of extracellular polysaccharides. A drastic increase in the total amount of EPS, as well as changes in EPS composition with increased protein amount, was also observed for *P. taiwanensis* VLB120ΔC biofilms grown in the presence of 0.5% (vol/vol) 1-butanol (32). In E. coli, the upregulation of membrane modification genes involved in exopolysaccharide synthesis (i.e., M-antigen or colanic acid) was observed in stress response to butanol, other industrially relevant organic solvents, and organic acids such as 2,4-butanediol and acetate, respectively (23). Therefore, as well established in *Bacteria* (56), alterations of the EPS composition and their component structures seems to be a typical response to environmental stresses, here exposure to 1-butanol, in S. acidocaldarius.

Transcriptome data support the observed switch from the planktonic to the biofilm mode of life. Archaella, the type IV pilus-like motility structures of *Archaea*, are involved in biofilm formation, species interactions, and adhesion (12, 35). All archaellum-encoding genes and the gene encoding ArnR1, the positive regulator of archaellum synthesis, were downregulated (35). Protein phosphorylation has been shown to play an important regulatory role in the transition from a motile (planktonic) to a sessile phenotype and thus biofilm formation (40, 42). For the Hanks-type protein kinase ArnC (Saci_1193) that phosphorylates the two negative regulators of motility (ArnA and ArnB), a slight upregulation was observed (40, 57). Also, the gene encoding the archaeal biofilm regulator 1 (AbfR1) was upregulated. Deletion of *abfR1* in S. acidocaldarius revealed a function in repression of EPS formation and activation of motility. However, in its phosphorylated form, AbfR1 was shown to support biofilm formation (41, 42). AbfR2, which was shown to enhance biofilm formation, was slightly downregulated (41, 43). These data suggest that major players involved in the complex regulatory network for motility and biofilm formation are affected by 1-butanol exposure in S. acidocaldarius. Notably, also in *Bacteria* such as E. coli, the downregulation of flagella and chemotaxis genes is reported in response to industrially relevant chemicals such as organic solvents and organic acids (23).

### Effect of 1-butanol on cell morphology.

We analyzed the cell morphology in response to 1-butanol using SEM. Besides the typical lobe-shaped cells with a smooth surface structure and flat cells with an irregular surface structure, we observed a third morphotype of more rounded cells with a perforated surface structure upon 1.5% (vol/vol) 1-butanol exposure. This is an interesting observation, since heterogeneity in archaeal cell communities has not been well addressed so far compared to *Bacteria* (58). Notably, the perforated morphology of S. acidocaldarius cells resembles SEM pictures of the Sulfolobus islandicus S-layer deletion strain (Δ*slaAB*) reported previously (59, 60). In the S-layer model, SlaB forms a stalk that anchors the cap (SlaA) in the cytoplasmic membrane and forms a crystalline proteinaceous matrix that covers the whole-cell surface. The Δ*slaA* strain was shown to lack the outermost lattice layer; the cells had an increased size and formed large aggregates called “bulky clumps” (60). An analysis of the chromosome content of single cells via flow cytometry revealed an uneven chromosome distribution and an increase of chromosome numbers in Δ*slaA* cells, suggesting a cell division defect in S. islandicus.

Both the removal of the S-layer as well as the addition of 1-butanol imply membrane stress. This is in line with our observation that planktonic cells in shaking cultures, where shear forces were applied, were more sensitive toward 1-butanol (complete growth inhibition at 1.5% [vol/vol]) than biofilm cells grown under static cultivation conditions (tolerance of up to 2.5% [vol/vol]). In accordance, we observed increased carbohydrate and protein concentrations in the EPS in response to 1-butanol exposure in our study. Furthermore, membrane proteins in general, as well as the *slaA* and *slaB* genes, were downregulated. In contrast, *cdvB1* and less-pronounced other genes of the ESCRT III machinery (except *cdvA* and *cdvB3*) were upregulated, suggesting an increased activity with possible function in vesicle formation, budding, and/or cytokinesis (36–39, 61). For the *cdvB* paralogs *cdvB1* and *cdvB2*, a function in ring formation and constriction during cell division, as well as vesicle formation, was shown, and for *cdvB1* a more important role under stress conditions was suggested (37, 39, 61).

Archaeal ESCRT III proteins have been identified in membrane vesicles excreted by *Sulfolobus* spp. (37). Vesicle formation is also well known for bacterial biofilms (62), where they serve various functions, including the transport of toxins, plasmid DNA, small RNA, quorum signaling molecules, and proteins. In addition, membrane vesicle formation was reported as a multiple-stress response mechanism that enhanced cell surface hydrophobicity and biofilm formation in Pseudomonas putida (63).

Notably, for some bacterial strains a change in cell morphology was also observed (32, 51, 52). A butanol-tolerant E. coli σ^70^ mutant showed an increased cell size and condensed cytoplasm, with occasionally invaginated bodies, in the presence of butanol (52). The inner membrane was still intact and not leaky upon butanol treatment, and the authors suggest a self-protection mechanism against damage from solvents.

### Effect of 1-butanol on cell protection mechanisms.

In response to 1-butanol exposure, we observed significant changes in the cells’ adaptive immunity system (CRISPR) and dormancy- or cell death-inducing defense (toxin-antitoxin) system. Many of the CRISPR-Cas system-related genes were downregulated in biofilms exposed to 1-butanol. The S. solfataricus CRISPR type III system has been studied (45, 64) and *in vitro* studies demonstrated RNA-degrading activity for Sso-IIIB (CMR), whereas Sso-IIID (CSM) cleaved both RNA and DNA (unspecific) (65). The gene encoding the Cas10 protein (*saci_1899*) was significantly downregulated. Cas10 catalyzes the formation of cyclic oligoadenylates, which act as second messengers activating defense mechanisms (66). The downregulation of the cells’ immune system and defense machinery (64) may increase the potential to acquire novel DNA. S. acidocaldarius*’* biofilm matrix (EPS) contains eDNA, which might enable DNA repair and horizontal gene transfer (13). However, alternative functions of CRISPR-Cas systems are well established for *Bacteria* in response to environmental stressors, such as nutrient starvation and iron limitation, and cell envelope stressors such as phage infection and high temperature (67). Furthermore, biofilm formation was shown to be regulated by the type I CRISPR-Cas system in P. aeruginosa (68).

In addition, significant changes in the regulation of the toxin-antitoxin (TA) system were observed (46). In Sulfolobus solfataricus, at least 26 virulence-associated protein (vap) *BC* TA loci (type II) were identified. Several *vapB-vapC* genes are activated by heat shock and the disruption of *vapB6* (antitoxin-encoding gene) resulted in susceptibility to thermal stress (69). TA systems are ubiquitous in prokaryotes (46). In *Bacteria,* they are supposed to provide a mechanism of cell persistence to cope with environmental stress (70). In B. subtilis biofilm formation, TxpA and YqcG toxins were shown to eliminate defective cells from developing biofilms upon nutrient starvation (71). Therefore, 1-butanol exposure in S. acidocaldarius seems to trigger changes in the adaptive immunity system (CRISPR) and dormancy- or cell death-inducing defense system (TA), supporting a functional coupling of both systems in order to allow for effective protection at the population level, as proposed previously by Makarova et al. (46).

### Effect of 1-butanol on cell metabolism and general stress response.

Finally, major transcriptional changes were also observed in metabolism. *Sulfolobus* spp. gain energy by aerobic respiration using a branched electron transport chain. Due to their acidophilic lifestyle (pH ∼3.0 outside the cell, pH ∼6.5 inside the cell) one of their major challenges is the maintenance of the intracellular pH, which is directly coupled to ATP generation via the proton motive force. In response to 1-butanol exposure, we observed significant changes in the transcription of components of the respiratory chain (i.e., the cytochrome *bc*_1_ complex and the DoxBCE complex). Therefore, as suggested previously for S. acidocaldarius in response to nutrient depletion (50), the differential regulation of the components of the respiratory chain seems to allow for adaptation to different stress conditions. The genes encoding 5-oxoprolinase, involved in the degradation of pyroglutamate, were the most upregulated genes in response to 1-butanol exposure in this study. Pyroglutamate is formed spontaneously under thermoacidophilic conditions by cyclization of glutamate. It can be used as the sole carbon source by S. acidocaldarius with 5-oxoprolinase as the key enzyme that catalyzes the ATP-dependent formation of glutamate (47). The upregulation of genes encoding proteins involved in the catechol pathway (*saci_2293* to *saci_2295*) indicates the degradation of aromatic amino acids (48). Hence, the exposure to 1% (vol/vol) 1-butanol resulted in obvious metabolic changes in respect to aerobic respiration and degradation of pyroglutamate and aromatic amino acids.

In addition, genes encoding peroxiredoxin, thioredoxin, and thioredoxin reductase were upregulated in response to 1-butanol. These serve as antioxidant proteins, eliminating reactive oxygen species (ROS) and thus protecting the cell from oxidative damage (72). In E. coli, *n*-butanol stress also resulted in the perturbation of respiratory complexes and a large increase of ROS (73).

In conclusion, S. acidocaldarius (shaking culture) showed a high tolerance for 1-butanol at up to 1% (vol/vol) at 76°C and pH 3.0. In response to solvent stress (i.e., 1-butanol, ethanol, 1-propanol, and isobutanol) we observed increased biofilm formation. For S. acidocaldarius biofilms, enhanced 1-butanol tolerance and changes in the EPS composition, biofilm architecture, and cell morphology with increased heterogeneity were observed. Finally, we analyzed the global response to solvent exposure at the gene and protein levels and identified significant changes, e.g., in motility, cell envelope, and the amount of membrane proteins, cell division, and vesicle formation, immune and defense systems, as well as metabolism and general stress response that are in line with the observed phenotypic characteristics. To our knowledge, this is the first detailed study on solvent stress response in a crenarchaeon, highlighting the impressive robustness of the thermoacidophilic S. acidocaldarius toward organic solvents.

## MATERIALS AND METHODS

### Strains and cultivation of liquid S. acidocaldarius cultures.

S. acidocaldarius strain DSM 639 was cultivated aerobically at 76°C in basal Brock medium, pH 3.0 (8), supplemented with 0.1% (wt/vol) NZ-amine (EZMix N-Z-Amine; Merck, Sigma-Aldrich, Darmstadt, Germany), 0.2% (wt/vol) d-glucose and different concentrations of 1-butanol (0% to 2.5% [vol/vol]; ≥99.5% p.a., Roth, Karlsruhe, Germany) or other organic solvents (ethanol, 99.9% GC, Fisher Scientific, Thermo Fisher Scientific, Waltham, MA, USA; 1-propanol, ≥99.5% GC, Merck; isobutanol, ≥99% GC, Honeywell Riedel de Haën, Fisher Scientific). Liquid cultures were incubated with agitation (180 rpm). Precultures of S. acidocaldarius (OD_600_ of 1.18, logarithmic phase) were used to inoculate fresh medium without or with 1-butanol (0% to 1.5% [vol/vol]) to a starting OD_600_ of 0.05. Cell growth (OD_600_ values) and concentrations of d-glucose and 1-butanol were determined regularly throughout 3 weeks of cultivation. Experiments were performed in four biological replicates.

### d-Glucose and 1-butanol quantification.

For 1-butanol or d-glucose quantification, a 1-ml aliquot of culture was removed at different time points (0 h, 48 h, 84 h, 168 h, and 336 h) and centrifuged at 16,000 × *g* for 10 min. Supernatants were stored at −20°C until use.

The d-glucose concentration was determined photometrically using glucose-6-phosphate dehydrogenase (G6PDH) from Thermotoga maritima following NADPH formation at 340 nm. The assay was performed in 100 mM HEPES/NaOH buffer at pH 6.5 and 70°C with 2 mM NADP^+^, 6 μl of G6PDH (after heterologous expression in Escherichia coli and purification by heat shock [20 min, 80°C]) and diluted culture supernatants (up to 25-fold) in a total volume of 500 μl. The reaction was started by the addition of sample. The d-glucose concentration was calculated using a standard calibration curve ranging from 0.04 to 0.5 mM d-glucose. The quantification of 1-butanol was performed enzymatically by use of alcohol dehydrogenase (ADH) from Saccharomyces cerevisiae (Merck, Sigma-Aldrich) following NADH formation. Briefly, the assay was conducted in sodium phosphate buffer (20 mM, pH 8.8) with 4 mM NAD^+^, 12 U ADH, and diluted culture supernatant in a total volume of 500 μl at 25°C. NADH formation was measured at 340 nm using a photometer (SPECORD 210; Analytik Jena, Jena, Germany). The 1-butanol concentrations were calculated using a standard calibration curve ranging from 0.05 to 1 mM 1-butanol.

### Cell aggregation analysis.

Cell aggregation was analyzed as described previously (74). Briefly, 5 μl of each culture (diluted to OD_600_ = 0.2) was spotted on microscope slides coated with 1% (wt/vol) agar. Cell aggregation was observed using a phase-contrast microscope with 100× magnification (Leica DMLS; Leica, Wetzlar, Germany).

### Spotting plates.

Samples of planktonic cell cultures were collected at different growth phases. Aliquots of 10 μl of the culture and dilutions (10^−1^ to 10^−6^ with Brock medium, pH = 5.0 to 5.5) were spotted on Brock-Gelrite plates containing 0.6 % gellan gum (Gelzan; Sigma-Aldrich), 0.1% (wt/vol) NZ-amine, and 0.2% (wt/vol) d-glucose. Plates were incubated at 76°C for 4 days. Then, growth was inspected and documented.

### Cultivation of S. acidocaldarius biofilms.

Brock medium, supplemented with 0.1% (wt/vol) NZ-amine, 0.2% (wt/vol) d-glucose, and different 1-butanol concentrations (0% to 2.5% [vol/vol]), was inoculated with an exponentially growing culture to an OD_600_ of 0.1 and grown for 4 days at 76°C in different incubation systems for biofilm formation. Growth (OD) was monitored at 600 nm.

**(i) Cultivation in 96-well microtiter plates.** To evaluate the solvent tolerance of S. acidocaldarius biofilms toward 1-butanol, cells were grown in the presence and absence of 1-butanol (see above, 150 μl total volume, 12 cavities per growth condition) in 96-well microtiter plates (Cell+, flat bottom, polystyrene; Sarstedt). Plates were sealed with a gas-impermeable aluminum foil (alu-sealing tape, pierceable; Sarstedt) and cultivated inside a metal box containing a water reservoir to reduce evaporation of the medium. After incubation, the OD_600_ was determined using a microplate reader (Infinite M200; Tecan). Afterwards, planktonic cells were transferred into a new microtiter plate and OD_600_ was determined again. The remaining biofilm biomass was quantified by crystal violet staining and its metabolic activity was analyzed using the resazurin assay.

**(ii) Cultivation on glass slides for light and scanning electron microscopy.** For light and scanning electron microscopy, biofilms were grown on sterile glass coverslips (18 mm × 18 mm; Roth) placed inside the wells of a 6-well microtiter plate (Cellstar; Greiner Bio-One International, Kremsmünster, Austria). An aliquot of 4.5 ml of an S. acidocaldarius culture was added to each well and then the plate was sealed with aluminum foil and incubated as described above. After incubation, planktonic cells were discarded, cavities were washed with 5 ml of Brock medium once, and biofilms were either stained with crystal violet for light microscopy or fixed for scanning electron microscopy.

**(iii) Cultivation in μ-dishes for confocal laser-scanning microscopy.** Aliquots of 4 ml of S. acidocaldarius cultures (0% and 1% [vol/vol] 1-butanol) were transferred into μ-dishes (ibi-treat, 35 mm, high; ibidi, Gräfelfing, Germany), sealed with aluminum foil, and incubated as described above.

**(iv) Cultivation in petri dishes for EPS extraction and transcriptome and proteome analyses.** Aliquots of 25 ml of S. acidocaldarius cultures (0%, 0.5%, and 1% [vol/vol] 1-butanol) were transferred into polystyrene petri dishes (92 × 16 mm, without cams; Sarstedt) and incubated for 4 days at 76°C in an air-tight chamber. For EPS extraction and transcriptomics and proteomics analyses, biofilms from 10 petri dishes (for each growth condition) were washed with 25 ml of Brock medium each, and collected from the bottom of the petri dishes by using a cell scraper.

### Crystal violet staining.

Crystal violet staining was used to visualize cells attached to abiotic surfaces after cultivation (75). The planktonic fraction of S. acidocaldarius liquid cultures was discarded and long-neck flasks were filled with 75 ml of a 0.01% (wt/vol) crystal violet (Merck) solution and incubated for 20 min. Afterward, the flasks were washed with ddH_2_O three times.

For light microscopy, biofilm on top of glass coverslips was stained with 0.01% (wt/vol) crystal violet solution for 20 min and washed with 5 ml ddH_2_O three times afterward.

For 96-well microtiter plates, 175 μl of a 0.1% (wt/vol) crystal violet solution was added into each cavity, incubated for 20 min, and washed three times with 200 μl ddH_2_O. After air drying, each well was filled with 200 μl of 95% (vol/vol) ethanol and the plates were incubated for 30 min to release the dye from attached biofilm into solution. The absorbance of crystal violet was measured at 570 nm using a microplate reader (Infinite M200; Tecan).

### Resazurin assay.

To analyze the metabolic activity of biofilm cells, a modified resazurin assay was established. After cultivation in microtiter plates (see above), biofilms were washed with 200 μl of Brock medium. Then 200 μl of 0.005% (wt/vol) resazurin (sodium salt; Sigma-Aldrich) in Brock medium (pH = 3.0) was added to each of the cavities. In an acidic milieu (pH = 3.0), the oxidoreduction dye resazurin is protonated to resorufin and exhibits a red color. Samples were incubated at 76°C for 3 h. By S. acidocaldarius respiratory activity, resorufin is converted to the colorless product dihydroresorufin. The conversion of resorufin was determined photometrically at 520 nm using a microplate reader (Infinite M200; Tecan). All experiments were performed in triplicates.

### Light microscopy.

For light microscopy, biofilms were grown on glass slides and stained with crystal violet as described above. After staining, glass slides were air dried and used for microscopy. Images were recorded using a light- and epifluorescence microscope and 4×, 40×, and 100× air objectives (Eclipse Ni; Nikon, Düsseldorf, Germany).

### Scanning electron microscopy.

For scanning electron microscopy (SEM), S. acidocaldarius biofilms were grown on glass slides (see above). Cells were fixed by the addition of 4 ml of 2% (vol/vol) glutardialdehyde in Brock medium and incubated for 2 h at 4°C. Then glass slides were submerged in acetone for 30 min. Biofilms were dried by critical point drying and sputtered with Au/Pd (80%/20%) for 15 to 30 s, resulting in a metal layer of 2.5 nm to 5.5 nm in thickness. Images were taken using a scanning electron microscope (QUANTA 400 FEG; FEI Company, Thermo Fisher Scientific).

### Confocal laser scanning microscopy.

After biofilm cultivation in μ-dishes (see above), the supernatant was removed carefully and 1 ml of Brock medium (pH = 7.0) was used to wash the submersed biofilm. For staining, 2 ml of a fluorescent staining solution containing 250 μM of the DNA-binding dye SYTO9 (excitation: 483 nm, emission: 503 nm; Invitrogen, Thermo Fisher Scientific), 7.5 μg/ml of the fluorophore-labeled lectin concanavalin A (ConA)-Alexa 633 (excitation: 632 nm, emission: 647 nm, α-mannopyranosyl and α-glucopyranosyl residues; Invitrogen), and 15 μg/ml of GS-IB4-Alexa 568 (isolectin from Griffonia simplicifolia, excitation: 578 nm, emission: 603 nm, α-d-galactosyl and *N*-acetyl-d-galactosamine residues; Invitrogen) in Brock medium (pH = 7.0) were used. Samples were incubated for 30 min in the dark at room temperature. After staining, the supernatant was removed, the biofilm was washed twice with 1 ml of Brock medium (pH = 7.0) and finally 2 ml of Brock medium was added. Visualization was performed using a Zeiss LSM 510 laser scanning microscope with a 100× oil objective. Data processing was performed using the software Imaris 8.1.2 (Bitplane, Zürich, Switzerland).

### Extraction of extracellular polymeric substances (EPS).

EPS extraction was performed using the cation-exchange resin (CER, Dowex Marathon C sodium form; Sigma-Aldrich) extraction method as described previously (14, 76). Briefly, biofilm cells were resuspended in phosphate buffer (2 mM Na_3_PO_4_·12 H_2_O, 4 mM NaH_2_PO_4_·1 H_2_O, 9 mM NaCl, 1 mM KCl, pH 7.0). After CER treatment of the biofilm suspension, centrifugation, and sterile filtration (14), the filtrate, corresponding to total extracellular material (TEM), contained cell-free low-molecular-weight compounds and EPS (of high molecular weight). To obtain EPS only, the filtrate was dialyzed against deionized water by use of a dialysis membrane with a molecular weight cutoff of 3.5 kDa (14).

### Quantification of EPS.

Carbohydrate and protein content were quantified in the biofilm suspension, and in the TEM and EPS solutions, as described previously (14). Proteins were quantified using a modified Lowry assay (77) with bovine serum albumin as a standard (Serva Electrophoresis, Heidelberg, Germany). Carbohydrates were quantified using the phenol-sulfuric acid method (78) and d-glucose as a standard for neutral carbohydrates.

### Determination of total cell counts.

Total cell counts in biofilm suspensions were determined by 4’,6-diamidino-2-phenylindole (DAPI; 5 μg/ml in 0.4% [vol/vol] formaldehyde) staining and filtration of the cells on polycarbonate filters (pore size 0.2 μm, 30 mm diameter; Merck). For microscopic cell counting, the filters were placed on a glass slide, covered with CitiFluor AF2 (Citifluor, Hatfield, PA, USA) and a coverslip. For statistic validity, 20 grids with 20 to 200 cells/grid were counted.

### Transcriptome analysis.

Biofilm and planktonic cell samples (supernatant after static incubation) were generated in petri dishes (92 × 16 mm, polystyrene, without cams; Sarstedt) as described before. Cells grown under six different conditions, including Biofilm-Control (BF0), Biofilm-0.5% (vol/vol) 1-Butanol (BF05), Biofilm-1% (vol/vol) 1-Butanol (BF1), Planktonic-Control (PL0), Planktonic-0.5% (vol/vol) 1-Butanol (PL05), and Planktonic-1% (vol/vol) and 1-Butanol (PL1), were separated, harvested by centrifugation (10 min, 5,000 × *g*, 4°C), and immediately frozen at −70°C. Isolation of cells was performed in triplicate with 10 technical replicas each. Samples were pooled to obtain sufficient cell mass for further processing. RNA was isolated using TRIzol (Thermo Fisher Scientific) as described previouslyl (79). The obtained RNA samples were purified as described by Bischof et al. (50). In accordance, sequencing libraries were prepared and quantified according to Bischof et al. Sequencing was performed on a MiSeq instrument (Illumina, San Diego, CA, USA) using v3 chemistry with a read length of 2 × 76 nucleotides (nt). Sequencing reads were mapped with Bowtie2 (80) against the reference genome of S. acidocaldarius DSM639. Mapped reads were counted and normalized as RPKM values (81) using the software ReadXplorer (82). In contrast to the original value, only reads mapping to coding sequences were considered for the calculation of the total number of mapped reads. For identification of differentially transcribed genes, the ratios (fold change) between the RPKM values obtained in different conditions for a single gene were calculated. Additionally, an A value (signal intensity value) was determined for all genes in each comparison (0.5 × log_2_ [RPKM condition1 × RPKM condition2]). Only genes with 4-fold (log_2_ fold change ≥2 or ≤−2) or 2-fold changes (log_2_ fold change ≥1 or ≤−1) (depending on the comparison) and an A value of ≥2 were considered differentially transcribed.

### Proteome analysis.

Biofilms were cultivated in petri dishes (92 × 16 mm, polystyrene, without cams, Sarstedt) and harvested as described above. Frozen cells of S. acidocaldarius, grown under two different conditions (Biofilm-Control [BF0] and Biofilm-1% [vol/vol] 1-butanol [BF1]), were washed twice with ice-cold water. Then protein extraction was performed as described by Bischof et al. (50). Briefly, cells were resuspended in protein extraction buffer and lysed using ultrasonic treatment. One hundred micrograms of protein of the supernatant (21,000 × *g* and 4°C for 30 min) was used for an iTRAQ (isobaric tags for relative and absolute quantitation) analysis with two 8-plex-iTRAQ tags 113 and 115 labeled for BF0 and BF1, respectively. The analysis was performed based on the manufacturer’s instruction (SCIEX, Framingham, MA, USA) as described in Bischof et al. (50). All raw data files from mass spectrometry (MS) analysis were submitted to MaxQuant version 1.5.3.8 for protein identification against the S. acidocaldarius database (consisting of 2,222 entries) downloaded in August 2015 from Uniprot (http://www.uniprot.org). All settings, data handling as well as quantitation, were performed according to Bischof et al. (50).

### Data availability statement.

RNA-seq data have been deposited in the ArrayExpress database at EMBL-EBI (www.ebi.ac.uk/arrayexpress) under accession number E-MTAB-10093. The mass spectrometry proteomics data have been deposited to the ProteomeXchange Consortium via the PRIDE (83) partner repository with the data set identifier PXD023858.

## Supplementary Material

Download

Download

Download
