# Supplementary material for: Exposure to 1-Butanol Exemplifies the Response of the Thermoacidophilic Archaeon Sulfolobus acidocaldarius to Solvent Stress
Source: Appl Environ Microbiol. 2021 May 11;87(11):e02988-20. doi: 10.1128/AEM.02988-20 (PMC8208165; doi:10.1128/AEM.02988-20)
Supplement: Download [file AEM.02988-20_aem.02988-20-s0001.pdf]

Supplementary Information

Table S1: Differentially regulated genes in *S. acidocaldarius* biofilm and planktonic cells grown statically in the presence and absence of 1% (v/v) 1-butanol (Petri dishes, 4d, 76 °C). The genes are ordered according to their involvement in cellular structures or processes. The effect of 1-butanol on gene expression in biofilm cells (BF1/BF0), planktonic cells (PL1/PL0) and the change in response to the respective lifestyle without 1-butanol exposure (BF0/PL0) is depicted. n. s.: not significantly regulated (log2 fold change < 1).

Locus	arCOG annotation	arCOG code	Biofilm (BF1/BF0) log2 fold change	Planktonic (PL1/PL0) log2 fold change	Lifestyle (BF0/PL0) log2 fold change
**Cell surface structures**					
**S-Layer**					
Saci_2354	S-layer protein SlaB	M	-1.58	n. s.	n. s.
Saci_2355	S-layer protein SlaA	M	-1.87	n. s.	n. s.
Saci_1846	Thermopsin-like protease	E	-1.90	-1.47	n. s.
**Pili**					
**UV induced pili**					
Saci_1493	Predicted component of type IV pili like system	N	n. s.	-1.36	n. s.
Saci_1494	ATPase involved in archaellum/pili biosynthesis	N	n. s.	n. s.	n. s.
Saci_1495	Pilus assembly protein TadC	N	n. s.	-1.31	n. s.
**Adhesive pili**					
Saci_2317	ATPase involved in archaellum/pili biosynthesis	N	n. s.	n. s.	n. s.
Saci_2318	Pilus assembly protein TadC	N	n. s.	n. s.	n. s.
Saci_2319	Pilin/Flagellin, FlaG/FlaF family	N	1.16	n. s.	-1.94
**Archaellum**					
Saci_1172	Archaellum assembly protein J, TadC family	N	-1.41	-2.16	n. s.
Saci_1173	ATPase involved in archaellum/pili biosynthesis	N	-2.32	-1.98	n. s.
Saci_1174	ATPase involved in biogenesis of archaellum	N	-3.12	-1.91	-1.03
Saci_1175	Archaellum protein F, archaellin of FlaG/FlaF family	N	-1.71	-2.90	n. s.
Saci_1176	Archaellum protein G, archaellin of FlaG/FlaF family	N	-2.71	-3.42	n. s.
Saci_1177	Component of archaellum, FlaD/E family	N	-2.33	-2.80	n. s.
Saci_1178	Archaeal flagellins	N	-2.79	-3.56	n. s.
**Vesicle/ESCRT system and Biofilm formation**					
**Vesicle/ESCRT system**					
Saci_0451	Archaeal division protein CdvB1, Snf7/Vps24/ESCRT-III family	D	2.14	1.51	-1.51
Saci_1372	Cell division ATPase of the AAA+ class, ESCRT system	D	1.36	n. s.	n. s.

Locus	arCOG annotation	arCOG code	Biofilm (BF1/BF0) log2 fold change	Planktonic (PL1/PL0) log2 fold change	Lifestyle (BF0/PL0) log2 fold change
	component, CdvC				
Saci_1373	Archaeal division protein CdvB, Snf7/Vps24/ESCRT-III family	D	1.26	n. s.	-1.19
Saci_1374	Archaeal division protein CdvA	D	n. s.	n. s.	n. s.
Saci_1416	Archaeal division protein CdvB2, Snf7/Vps24/ESCRT-III family	D	1.41	n. s.	-1.82
Saci_1601	Archaeal division protein CdvB3, Snf7/Vps24/ESCRT-III family	D	n. s.	n. s.	n. s.
**Glycosyltransferases**					
Saci_1914	Glycosyltransferase	M	-1.87	-1.21	n. s.
Saci_1915	Glycosyl transferase family 2	M	-1.15	-1.06	n. s.
Saci_1923	Glycosyltransferase	M	-1.05	n. s.	n. s.
Saci_1926	Glycosyl transferase family 2	M	n. s.	n. s.	-1.90
**Regulation and stress response**					
**Transcriptional regulators**					
Saci_0102	Transcriptional regulator, contains HTH domain	K	-1.05	n. s.	n. s.
Saci_0446	Transcriptional regulator, contains HTH domain (AbfR1)	K	1.71	n. s.	n. s.
Saci_0665	Homolog of transcription initiation factor TFIIB, contains Zn-ribbon domain	K	n. s.	-1.41	-1.03
Saci_1171	Predicted transcriptional regulator (ArnR1)	K	-1.62	-2.01	n. s.
Saci_1209	Transcriptional regulator, contains HTH domain	K	1.17	n. s.	n. s.
Saci_1223	Transcriptional regulator, contains HTH domain (AbfR2)	K	-1.54	n. s.	n. s.
Saci_1588	DNA-binding transcriptional regulator, Lrp family	K	n. s.	1.73	n. s.
Saci_1992	CRISPR-Cas associated transcriptional regulator, contains HTH domain, lacking CARF domain	K	-3.19	-2.35	1.01
**Protein phosphorylation: protein kinases and phosphatases**					
Saci_0545	Protein-tyrosine phosphatase (PTP)	T	-1.30	n. s.	n. s.
Saci_0796	RIO-like serine/threonine protein kinase fused to N-terminal HTH domain (RIO2)	T	n. s.	-1.09	n. s.
Saci_1181	Membrane associated serine/threonine protein kinase (ArnS)	R	-1.19	-1.77	n. s.
Saci_1193	Membrane associated serine/threonine protein kinase (ArnC)	R	1.22	n. s.	-1.65

Locus	arCOG annotation		arCOG code	Biofilm (BF1/BF0) log2 fold change	Planktonic (PL1/PL0) log2 fold change	Lifestyle (BF0/PL0) log2 fold change
**CRISPR-Cas**						
**Type I-D system**						
Saci_1864	CRISPR-Cas system related protein, RAMP superfamily Cas6 group	cas6	V	n. s.	n. s.	n. s.
Saci_1872	CRISPR-Cas system related helicase, Cas3 (C-terminal HD nuclease domain)	cas3	V	n. s.	n. s.	n. s.
Saci_1873	CRISPR associated protein, RAMP family Cas5 group	csc1gr5	V	n. s.	n. s.	n. s.
Saci_1874	CRISPR-Cas system related protein, RAMP superfamily Cas7 group	csc2gr7	V	n. s.	n. s.	n. s.
Saci_1875	CRISPR associated protein Cas10d, large subunit of Type I-D system effector complex, contains HD family nuclease	cas10d	V	n. s.	n. s.	n. s.
Saci_1876	CRISPR-Cas associated transcriptional regulator, contains CARF and HTH domain	casR	VK	-1.23	n. s.	n. s.
Saci_1877	CRISPR-Cas system related protein, RAMP superfamily Cas6 group	cas6	V	-1.56	n. s.	n. s.
Saci_1879	CRISPR-associated protein Cas2	cas2	V	n. s.	n. s.	n. s.
Saci_1880	CRISPR-associated protein Cas4	cas4	V	n. s.	n. s.	n. s.
Saci_1881	CRISPR-associated protein Cas1	cas1	V	-1.54	n. s.	n. s.
***Sulfolobus* specific Type III system**						
Saci_1893	CRISPR-Cas system related protein, RAMP superfamily Cas7 group	csm3gr7	V	n. s.	n. s.	n. s.
Saci_1896	CRISPR-Cas system related protein, RAMP superfamily Cas7 group	csm3gr7	V	-1.14	n. s.	n. s.
Saci_1897	CRISPR associated protein, possible subunit of Type III-A	csx26	V	-2.07	n. s.	1.21

Locus	arCOG annotation		arCOG code	Biofilm (BF1/BF0) log2 fold change	Planktonic (PL1/PL0) log2 fold change	Lifestyle (BF0/PL0) log2 fold change
	effector complex					
Saci_1898	CRISPR associated protein, Csm4g5-like subunit of effector complex	csm4 gr5	V	-2.12	n. s.	1.37
Saci_1899	CRISPR associated protein, Cas10-like subunit Type III-A effector complex	cas10	V	-2.60	-1.14	1.02
**Adaptation/processing module**						
Saci_2008	CRISPR-Cas system related protein, RAMP superfamily Cas6 group	cas6	V	-3.02	-1.44	n. s.
Saci_2010	CRISPR-associated protein Cas2	cas2	V	-1.83	n. s.	n. s.
Saci_2011	CRISPR-associated protein Cas1	cas1	V	-1.40	n. s.	n. s.
Saci_2012	CRISPR-associated protein Cas4	cas4	V	n. s.	n. s.	n. s.
**Type III-D system**						
Saci_2043	CRISPR-Cas system related protein, RAMP superfamily Cas7 group	csm3 gr7	V	n. s.	n. s.	n. s.
Saci_2044	CRISPR-associated protein, RAMP family Cas5 group, signature protein for Type III-D system	csx10 gr5	V	n. s.	n. s.	n. s.
Saci_2045	CRISPR-Cas system related protein, RAMP superfamily Cas7 group	csm3 gr7	V	n. s.	n. s.	-1.09
Saci_2046	CRISPR associated protein, Cas10-like subunit Type III-A effector complex	cas10	V	-2.42	n. s.	n. s.
Saci_2048	CRISPR-Cas system related protein, RAMP superfamily Cas7 group	csm3 gr7	V	n. s.	n. s.	n. s.
Saci_2049	CRISPR-Cas system related protein, RAMP superfamily Cas7 group	csm3 gr7	V	-2.02	n. s.	n. s.
Saci_2052	CRISPR-associated protein	csm2 gr11	V	1.22	1.03	-1.63

Locus	arCOG annotation	arCOG code	Biofilm (BF1/BF0) log2 fold change	Planktonic (PL1/PL0) log2 fold change	Lifestyle (BF0/PL0) log2 fold change
**Toxin-Antitoxin**					
Saci_0264	Transcriptional regulator, CopG/Arc/MetJ family (DNA-binding and a metal-binding domains)	V	1.02	-1.36	n. s.
Saci_0322	CopG/RHH family DNA binding protein, antitoxin	V	1.02	n. s.	n. s.
Saci_0942	CopG/MetJ, RHH domain containing DNA-binding protein, often an antitoxin in Type II toxin-antitoxin systems	V	n. s.	-1.71	n. s.
Saci_1056	Antitoxin, CopJ/RHH family	V	-2.03	-4.14	n. s.
Saci_1124	CopG/RHH family DNA binding protein	V	-2.03	n. s.	n. s.
Saci_1812	RHH/CopG DNA binding protein	V	-1.44	-1.69	n. s.
Saci_1928	Minimal nucleotide transferase MNT, antitoxin of HEPN-MNT system	V	1.28	1.32	
Saci_1932	RHH/copG family antitoxin	V	-1.57	-1.36	n. s.
Saci_1936	RHH/CopG DNA binding protein	V	-1.53	-1.38	n. s.
Saci_1947	RHH/CopG DNA binding protein	V	n. s.	-1.11	n. s.
Saci_1952	CopG/RHH family DNA binding protein, antitoxin	V	n. s.	-1.40	n. s.
Saci_1980	RHH/CopG DNA binding protein	V	n. s.	-1.34	n. s.
Saci_2003	CopG/RHH family DNA binding protein, antitoxin	V	n. s.	n. s.	n. s.
Saci_2079	RHH/CopG DNA binding protein	V	n. s.	2.19	1.23
**Metabolism**					
**Amino acid metabolism**					
**Pyroglutamate conversion**					
Saci_2041	N-methylhydantoinase A/5-oxoprolinase, beta subunit	E	4.33	3.73	-1.28
Saci_2042	N-methylhydantoinase B/5-oxoprolinase, alpha subunit	E	4.15	3.68	-1.16
Saci_2036	N-methylhydantoinase A/5-oxoprolinase, alpha subunit	E	3.40	2.19	-2.30
**Aromatic compound/amino acid conversion**					
Saci_2293	2-keto-4-pentenoate hydratase/2-oxohepta-3-ene-1,7-dioic acid hydratase (catechol pathway)	Q	3.17	2.70	n. s.
Saci_2294	4-hydroxyphenylacetate 3-monooxygenase	Q	3.14	2.41	n. s.
Saci_2295	Catechol 2,3-dioxygenase or other lactoylglutathione lyase family enzyme	E	3.39	2.49	n. s.

Locus	arCOG annotation	arCOG code	Biofilm (BF1/BF0) log2 fold change	Planktonic (PL1/PL0) log2 fold change	Lifestyle (BF0/PL0) log2 fold change
**Antioxidance defence**					
Saci_1125	peroxiredoxin	O	1.20	2.13	n. s.
Saci_1169	thioredoxin reductase	O	2.61	3.08	n. s.
Saci_1823	thioredoxin	O	1.37	2.71	n. s.
**Respiratory chain**					
**Cytochrome bc1 complex (SoxNL-CbsAB-OdsN)**					
Saci_1859	Cytochrome b558/566, subunit B	C	-3.70	-3.14	1.10
Saci_1860	Rieske Fe-S protein	C	-1.66	n. s.	n. s.
Saci_1861	Cytochrome b subunit of the bc complex	C	-1.94	-1.25	n. s.
Saci_1862	Heme-degrading monooxygenase HmoA and related ABM domain proteins	H	-1.50	n. s.	n. s.
**Terminal oxidase SoxABCDL complex (Saci_2086-2089): Not significantly regulated**					
**Terminal oxidase SoxEFGHIM complex**					
Saci_2258	Predicted subunit of heme/copper-type cytochrome/quinol oxidase	C	n. s.	-1.36	n. s.
Saci_2259	Heme/copper-type cytochrome/quinol oxidase, subunit 2	C	1.95	n. s.	-1.16
Saci_2260	Cytochrome b subunit of the bc complex	C	n. s.	-2.20	-1.22
Saci_2261	Rieske Fe-S protein	C	-1.13	-2.27	n. s.
Saci_2262	Sulfocyanin	C	n. s.	-1.98	-1.41
Saci_2263	Heme/copper-type cytochrome/quinol oxidase, subunit 1 and 3	C	n. s.	-1.53	n. s.
**Terminal oxidase DoxBCE complex**					
Saci_0097	Heme/copper-type cytochrome/quinol oxidase, subunit 1	C	-2.45	-1.94	n. s.
Saci_0098	Terminal oxidase, subunit doxC	C	-2.79	-1.69	n. s.
Saci_0099	Terminal oxidase, subunit doxE	C	-3.19	-1.24	n. s.

Table S2: Highly downregulated genes encoding for membrane proteins in static grown *S. acidocaldarius* biofilm cells in response to 1-butanol (1% (v/v) exposure (static cultivation in Petri dishes, 4d, 76 °C).

**Locus**	**arCOG annotation**	**arCOG functional code**	**Regulation (BF1/BF0)**		**Regulation (PL1/PL0)**	
			log2 fold change	A-value	log2 fold change	A-value
Saci_0301	uncharacterized membrane protein, DUF981 family	S	-8.32	14.11	-7.13	12.82
Saci_1074	uncharacterized membrane protein	S	-7.06	13.43	-3.93	12.80
Saci_1753	uncharacterized membrane protein, virus associated	X	-5.98	11.62	-2.48	11.76
Saci_0516	uncharacterized protein	S	-5.28	3.06	-2.71	3.69

Table S3: Significantly regulated proteins in static grown *S. acidocaldarius* biofilm cells in response to 1-butanol (1% (v/v) exposure (static cultivation in Petri dishes, 4d, 76 °C).

**Locus**	**arCOG annotation**	**arCOG functional code**	**log2 (BF1/BF0)**
Saci_0642	Ribosomal protein L37E	J	1.72
Saci_0843	Transcriptional regulator, contains N-terminal RHH domain	K	1.47
Saci_0855	Zn-ribbon protein	S	1.19
Saci_0345	Lipoate-protein ligase A	H	1.16
Saci_0107	Molybdopterin-guanine dinucleotide biosynthesis protein	H	1.14
Saci_1468	DNA-binding TFAR19-related protein, PDSD5 family	R	1.11
Saci_0583	Ribosomal protein S14	J	1.10
Saci_0356	Uncharacterized small metal-binding protein	S	1.04
Saci_1079	Threonine dehydrogenase or related Zn-dependent dehydrogenase	E	1.04
Saci_0182	Prephenate dehydrogenase	E	1.03
Saci_1261	Threonyl-tRNA synthetase	J	1.02
Saci_2322	Cobalamin biosynthesis protein CbiG	H	-1.01
Saci_1208	Predicted dithiol-disulfide isomerase involved in polyketide biosynthesis	Q	-1.05
Saci_1366	Uncharacterized protein	S	-1.09
Saci_1764	ABC-type dipeptide/oligopeptide/nickel transport system, ATPase component	E	-1.10
Saci_2119	RecB family nuclease with coiled-coil N-terminal domain	R	-1.10
Saci_1306	Uridylate kinase	F	-1.14
Saci_1308	Short-chain alcohol dehydrogenase	I	-1.15

Locus	arCOG annotation	arCOG functional code	log2 (BF1/BF0)
Saci_0319	Uncharacterized protein YjgD, DUF1641 family	S	-1.15
Saci_1168	Ser-tRNA(Ala) deacylase AlaX (editing enzyme)	J	-1.18
Saci_0845	Uncharacterized protein	S	-1.20
Saci_0820	Riboflavin synthase beta-chain	H	-1.20
Saci_0177	Single-stranded DNA-specific exonuclease RecJ	L	-1.22
Saci_1862	Heme-degrading monooxygenase HmoA and related ABM domain proteins	H	-1.24
Saci_1633	Enoyl-CoA hydratase/carnithine racemase	I	-1.27
Saci_0668	Uncharacterized protein	S	-1.27
Saci_0415	Zn-dependent protease with chaperone function	O	-1.30
Saci_0097	Heme/copper-type cytochrome/quinol oxidase, subunit 1	C	-1.35
Saci_1243	Uncharacterized protein	S	-1.36
Saci_2355	S-layer protein SlaA	M	-1.39
Saci_2332	Membrane protease subunit, stomatin/prohibitin homolog	O	-1.48
Saci_2139	CBS domain containing protein	R	-1.54
Saci_1250	Glycosyl hydrolase family 15	G	-1.74
Saci_1860	Rieske Fe-S protein	C	-1.91

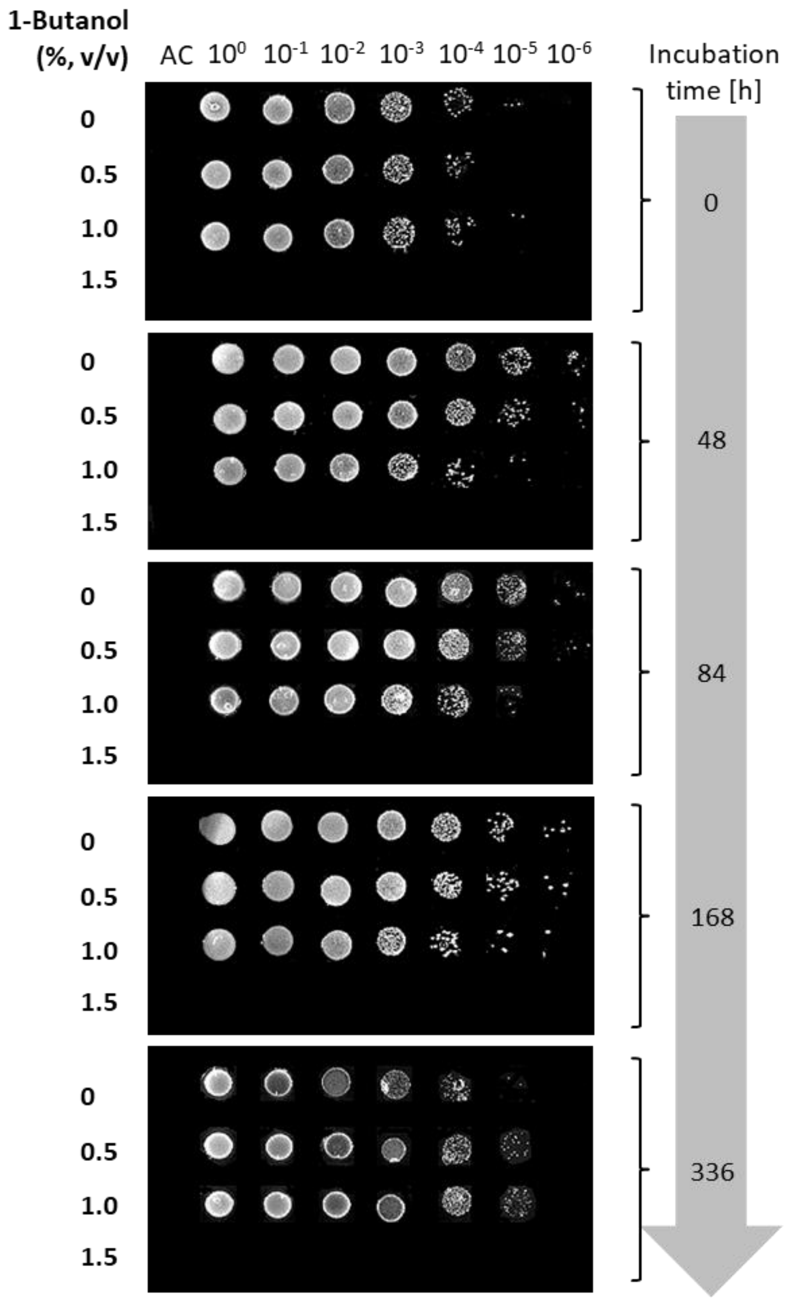

Figure S1: Culturability of *S. acidocaldarius* DSM 639 after 1-butanol exposure.

S. acidocaldarius DSM 639 liquid cultures were exposed to different concentrations of 1-butanol (0% to 1.5% (v/v)) in Brock medium supplemented with 0.1% (w/v) NZ-amine and 0.2% (w/v) D-glucose. After different cultivation times (0, 48, 84, 168 and 336 h), 10 µl of undiluted culture (10^0) or diluted culture (10^{-1}-10^{-6}) were spotted on Brock-Gelrite plates (0.1% (w/v) NZ-amine, 0.2% (w/v) D-glucose). Spot plates were incubated at 76 °C for four days. An abiotic control (AC, medium without cells) and the 10^0-10^{-6} 10-fold dilution series of *S. acidocaldarius* DSM 639 shaking cultures are shown.

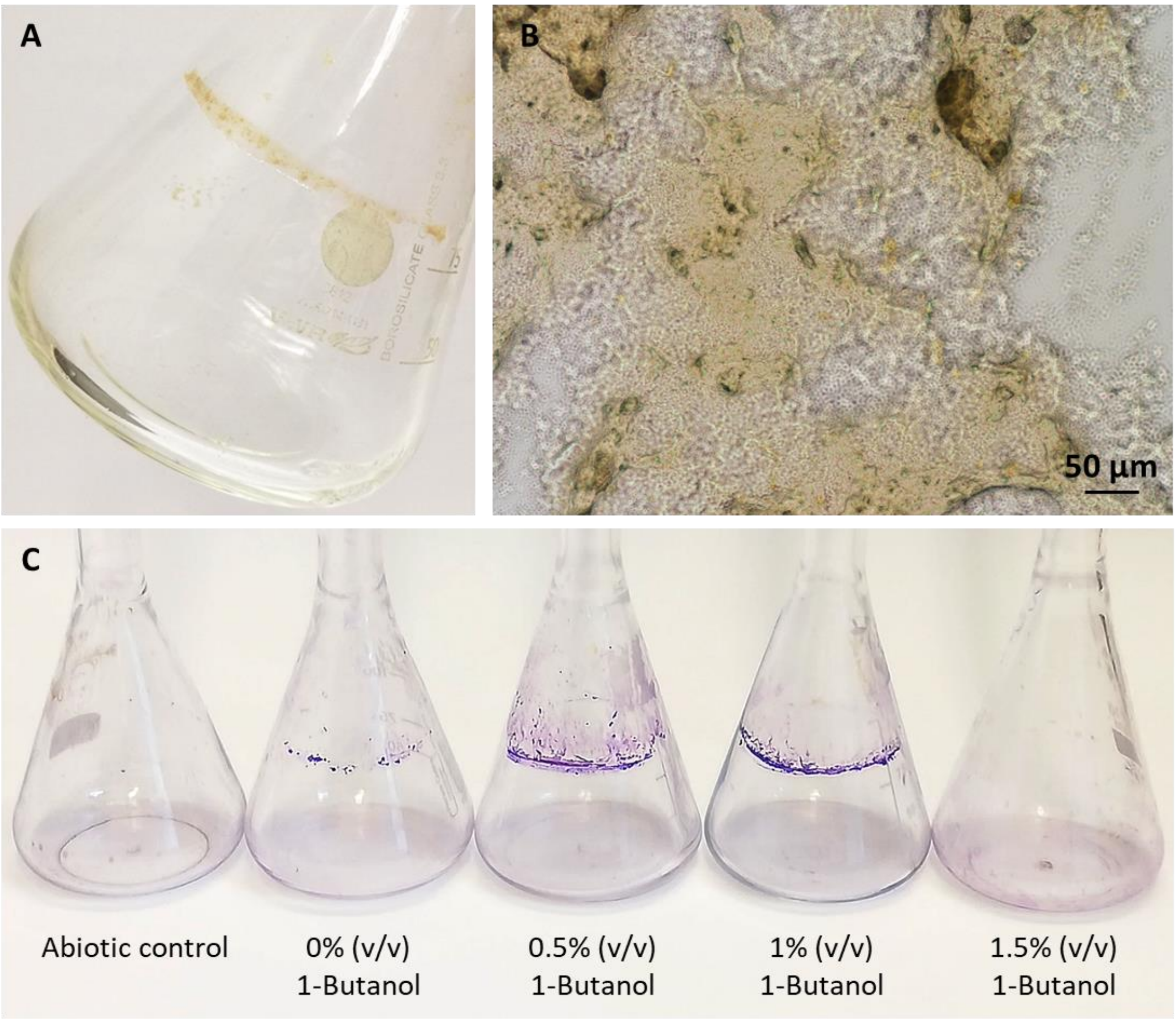

Figure S2: Adhesion of *S. acidocaldarius* DSM 639 cells after cultivation with 0.5% (v/v) and 1% (v/v) 1-butanol. Slimy material was observed at the liquid-air interfaces on the glass surface of planktonic *S. acidocaldarius* DSM 639 cultures exposed to 0.5% (v/v) and 1% (v/v) 1-butanol (Brock medium, 0.1% NZ-amine and 0.2% D-glucose). **A**. Collar of slimy material inside the Erlenmeyer flask of *S. acidocaldarius* DSM 639 culture exposed to 1% (v/v) 1-butanol. Culture fluid was discarded. The visible material was scrabbed off the glass surface using a cell scraper, applied on a cavity slide and used for light microscopy (**B**). Large aggregates of organic material surrounding *S. acidocaldarius* cells were visible. **C**. After three weeks of cultivation biofilm formation of planktonic *S. acidocaldarius* DSM 639 cultures exposed to 0% to 1.5% (v/v) 1-butanol was visualized by crystal violet staining. For biofilm visualization cultures fluid was discarded, the empty Erlenmeyer flasks were stained with 0.01% (w/v) crystal violet solution and washed with water. Experiments were carried out in three to four biological replicates.

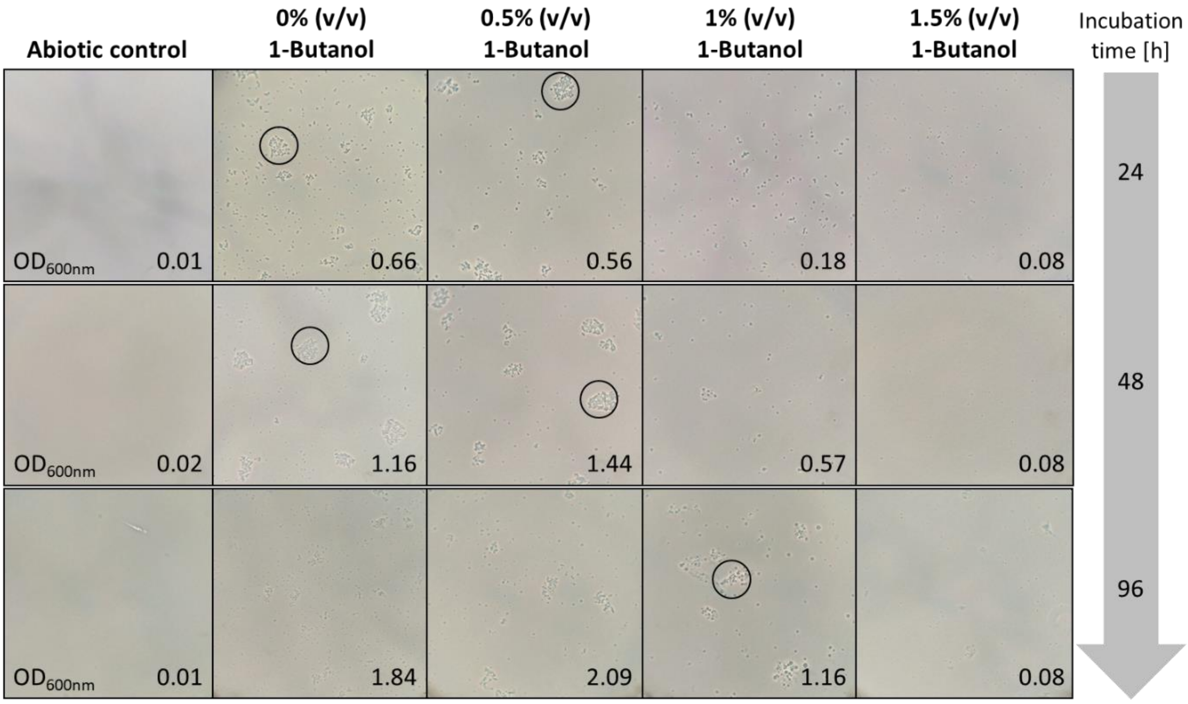

Figure S3: Cell aggregation analysis of *S. acidocaldarius* DSM 639 after 1-butanol exposure. Phase-contrast microscopy images of *S. acidocaldarius* DSM 639 shaking cultures exposed to different concentrations of 1-butanol (0% to 1.5% (v/v)) in Brock medium supplemented with 0.1% NZ-amine and 0.2% D-glucose. Circles mark examples of cell aggregates. OD_{600nm}: optical density at 600 nm.

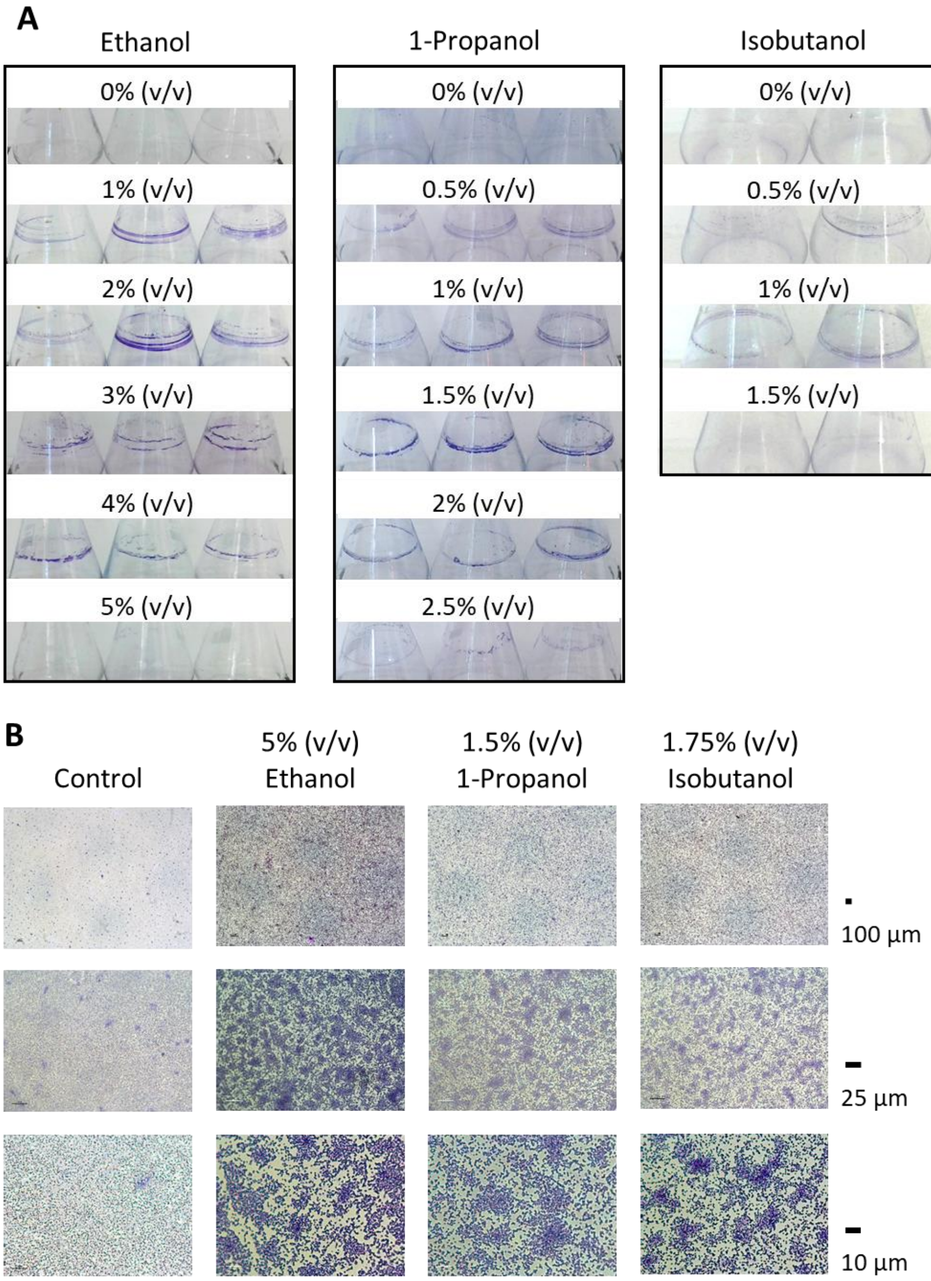

Figure S4: Effect of organic solvents on *S. acidocaldarius* cell adhesion and cell distribution. **A**. Cell adhesion of *S. acidocaldarius* DSM 639 after cultivation with different concentrations of ethanol, 1-propanol and isobutanol. Biofilms were visualized using crystal violet staining. The presence of multiple "collars" of the slimy material inside the flasks was presumably caused by medium loss due to sampling and medium evaporation, resulting in slightly decreasing culture volumes inside the flasks during the three weeks of the growth experiments. **B**. Effect of ethanol, 1-propanol and isobutanol exposure on *S. acidocaldarius* cell distribution. *S. acidocaldarius* was grown on glass surfaces for 4 d at 76 °C in presence and absence of different organic solvents. Biofilms were stained by crystal violet for subsequent analysis by light microscopy.

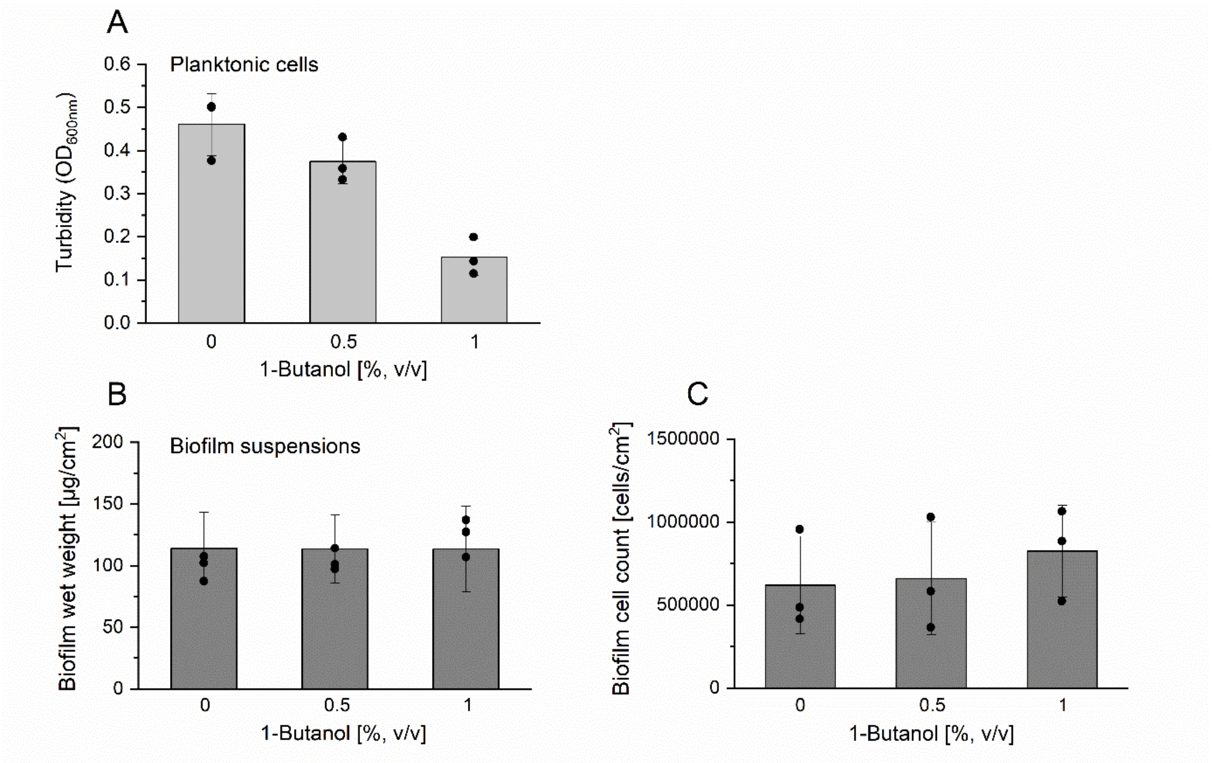

Figure S5: Influence of 1-butanol on biofilm formation. The amounts of planktonic (**A**) and biofilm cells of *S. acidocaldarius* (**B, C**) grown statically in Petri dishes were determined. Cultures were incubated at 76 °C for four days. **A**. Growth of planktonic cells was determined by turbidity measurements (OD_{600nm}; n = 3). **B**. Biofilm wet weight. The biomass was isolated from the bottom of Petri dishes for each condition and pooled biofilm samples were weighed (n = 3). **C**. Total cell counts of biofilm suspensions. Cell count was determined using the DAPI staining method (n = 3).

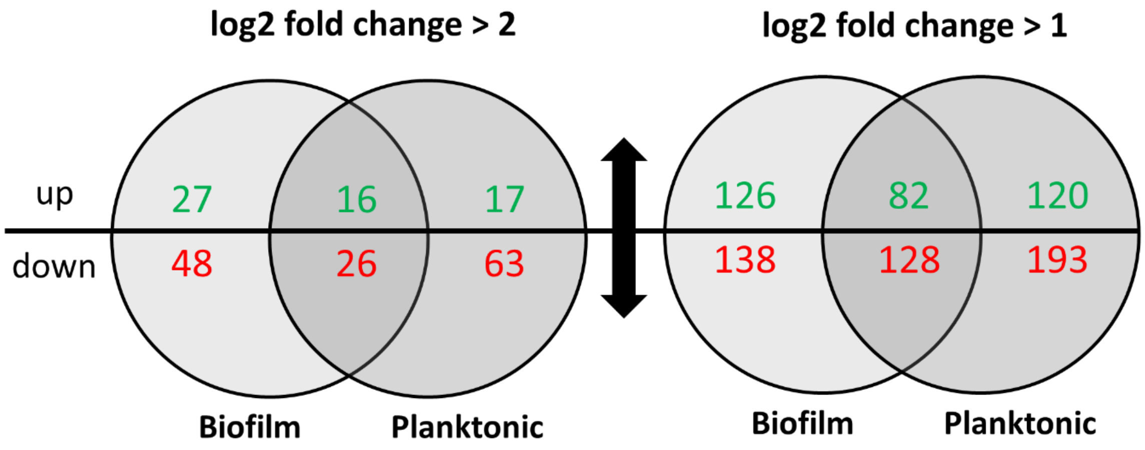

Figure S6: **Venn diagram displaying the overlap of significantly regulated genes in response to 1% (v/v) 1-butanol in biofilm and planktonic cells**. The numbers of log2 fold change > 2 and log2 fold change > 1 upregulated (green) and downregulated (red) genes are given. Intersections present the numbers of genes that are commonly regulated in both lifestyles.

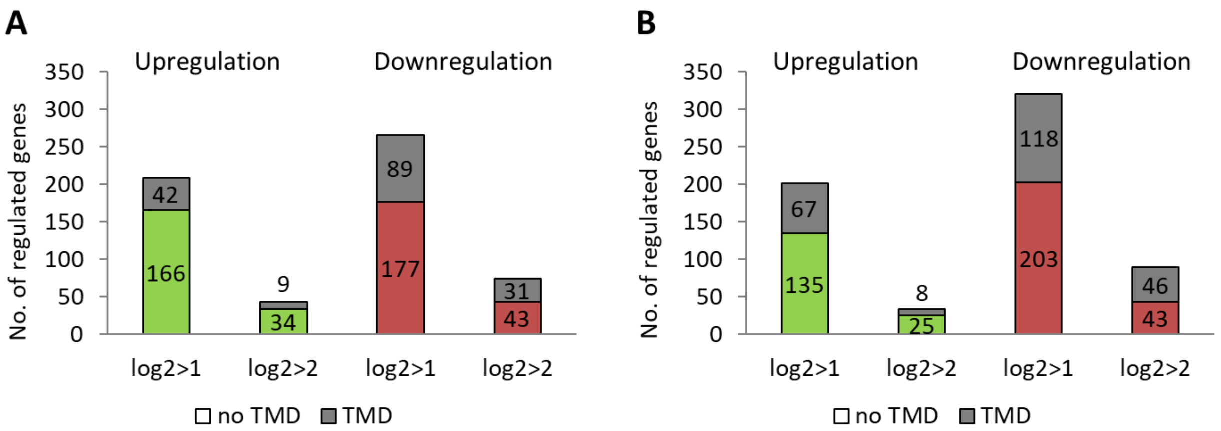

Figure S7: **Number of regulated genes encoding proteins without or with trans-membrane domains (TMD) in biofilm (A) and planktonic lifestyle (B).** The absolute numbers of >log2 fold change = 1 and >log2 fold change = 2 up- or downregulated genes are given for each lifestyle (green or red, respectively).
